# Quality of life item banks for type 2 diabetes: psychometric evaluation and computerized adaptive testing simulations

**DOI:** 10.1007/s11136-026-04387-6

**Published:** 2026-09-21

**Authors:** Eva K. Fenwick, Ryan E.K. Man, Aricia X.Y. Ho, Ester P.X. Lee, E. Shyong Tai, Ngiap Chuan Tan, Sue-Anne E.S. Toh, Tat Yean Tham, Chin Meng Khoo, Yong Mong Bee, Ecosse L. Lamoureux

**Affiliations:** 1 Singapore Eye Research Institute and Singapore National Eye Centre, The Academia, 20 College Road, Level 6, 169856 Singapore, Singapore; 2 Duke–NUS Medical School, National University of Singapore, Singapore, Singapore; 3 Department of Medicine, Yong Loo Lin School of Medicine, National University of Singapore, Singapore, Singapore; 4 SingHealth Polyclinics, 167 Jalan Bukit Merah Connection One (Tower 5), #15-10, Singapore, 150167 Singapore; 5 Clinical Affairs Department, Frontier Healthcare Group, Singapore, Singapore; 6 Division of Endocrinology, Department of Medicine, National University Hospital, Singapore, Singapore; 7 Department of Endocrinology, Singapore General Hospital, Singapore, Singapore; 8 Department of Surgery, The University of Melbourne, Melbourne, Australia

**Keywords:** Type 2 diabetes, Quality of life, Item banks, Rasch analysis, Computerized adaptive testing

## Abstract

**Purpose:**

We evaluated the psychometric properties of type 2 diabetes (T2DM) quality of life (QoL) item banks (IBs), and assessed their efficiency for computerized adaptive testing (CAT) using simulations.

**Methods:**

In this cross-sectional, clinical study, patients with T2DM answered 256 items across seven IBs: Activity Limitation (AL); Symptoms (SY); Diet (DT); Emotional (EM); Concerns (CN); DM Management (DMn); and Work (WK), referred to collectively as “DiabCAT”. The psychometric properties of each IB (e.g. measurement precision; item “fit”; differential item functioning) were assessed using Rasch analysis. The mean number of items required for ‘high’ (0.3 standard error [SE]) and ‘moderate’ (0.387 SE) precision was determined using CAT simulations.

**Results:**

The 298 patients (57.4% male) had a mean (SD) age of 60.7 (10.8) years and DM duration of 15.3 (9.7) years. Overall, SY, DT, WK, EM, and CN displayed acceptable measurement performance after deleting 15 problematic items, with some limitations in targeting at higher levels of the latent trait, particularly for EM. The DMn domain was multidimensional and subsequently split into two unidimensional domains (DMn-Convenience [DMn-CV] and DMn-Concerns [DMn-CN]). Owing to unresolvable psychometric issues, AL was dropped. In CAT simulations, the mean number of items required per IB ranged from 9 (CN, DMn-CV, WK) to 11 (EM) for moderate, and 14 (DT) to 18 (EM) for high measurement precision.

**Conclusions:**

Comprising 213 items within seven psychometrically valid and efficient IBs, DiabCAT enables researchers and clinicians to comprehensively assess the QoL impact of T2DM and its management with relatively low administration burden. Further assessment of its reliability, validity and responsiveness is warranted.

**Supplementary Information:**

The online version contains supplementary material available at https://doi.org/10.1007/s11136-026-04387-6.

## Introduction

Diabetes mellitus (DM) is a global health concern, with an estimated 630 million people expected to have the condition by 2045, mostly comprising Type 2 DM (T2DM) [1]. Singapore has the second highest prevalence of T2DM (~ 13%) among developed nations and [2], by 2050, 50% of Singaporeans aged ≥ 70 years may be affected [3]. T2DM requires a high degree of self-management and complex treatment regimens to maintain health and lower risk of developing disabling vascular complications, often resulting in reduced quality of life (QoL), including symptoms, mental health distress, and financial insecurity [4, 5].

A comprehensive understanding of the impact of T2DM on patients’ QoL and the patient-centred effectiveness of treatment is crucial in value-based care models [6]. Similarly, the Food and Drug Administration (FDA) now requires patient-centred endpoints to be incorporated into clinical trials [7]. Many generic (e.g., Short Form-36 [SF-36] [8]) and DM-specific (i.e., Problem Areas in Diabetes scale [PAID] [9] and Audit of Diabetes-Dependent Quality of Life [ADDQoL]) [10] patient-reported outcome measures (PROMs) are available to assess QoL outcomes in those with DM. However, limitations with these PROMs have been identified, including low patient-specific input during development, suboptimal validity indices, and lack of responsiveness data [4, 11–14]. Similarly, patients must respond to all questions even if they are too hard or too easy, which can increase test-taking burden. Some DM-specific PROMs also measure only single domains (i.e., distress [15], symptoms [16]) to avoid being too lengthy.

Item banking (IB) and computerized adaptive testing (CAT) address many of these issues [17]. An IB is a collection of items that measure a single latent construct, calibrated on an interval-level scale using modern psychometric methods [17]. A CAT algorithm administers items that are most informative at each point in the test until a stopping criterion (e.g. measurement precision, based on standard error of measurement [SE]) is reached [18]. As item administration is targeted to the patients’ level of the construct, CATs are generally shorter than fixed-length PROMs and provide an efficient way of estimating relevant QoL outcomes [17, 18]. Although Patient-Reported Outcomes Measurement Information System (PROMIS) generic CATs (e.g. Physical Function, Pain Interference, Sleep Disturbance, Anxiety, Depression, and Ability to Participate in Social Roles and Activities) have been validated in some populations with DM [19], their item content was not developed specifically for individuals living with DM. Consequently, these measures may lack sensitivity to DM-specific experiences and may not fully capture the unique challenges associated with managing this condition. Based on a comprehensive literature review, and qualitative interviews with patients and diabetes specialists [20], our team has developed a 7-domain, 256-item T2DM-specific QoL PROM (“DiabCAT”). Here, we evaluate the psychometric properties of the DiabCAT IBs using data from a clinical sample of patients with T2DM, and assess CAT performance using simulations.

## Methods

### Sample population

Patients aged ≥ 21 years (English- or Mandarin-speaking) with a clinical diagnosis of T2DM were recruited from the medical retina and endocrinology clinics at the Diabetes & Metabolism Centre at the Singapore General Hospital (October 2023 to June 2025). Patients were excluded if they had Type 1 or gestational diabetes, other non-diabetes related co-morbidities significantly affecting QoL (e.g. late-stage cancer), cognitive impairment (assessed by the 6-item Cognitive Impairment Test [6-CIT] [21]) or hearing impairment precluding them from hearing the questions. Patients’ medical and pharmaceutical records were screened by a Clinical Research Coordinator (CRC) to identify individuals with T2DM who matched our eligibility criteria (Additional File 1). We recruited patients with differing ethnicities, genders, ages, levels of glycaemic control, DM duration, complications and treatment modalities to ensure our calibrations were broadly applicable.

The study protocol was approved by the SingHealth Centralised Institutional Review Board (CIRB #2021/2482) and written informed consent was obtained from participants. The study was conducted in accordance with the Declaration of Helsinki.

### Development of the T2DM IBs

DiabCAT’s domain and item development process has been described previously [20]. In brief, following qualitative input from 42 patients with T2DM and eight DM-specific healthcare practitioners, the DiabCAT instrument comprised 256 items within seven QoL domains: Symptoms (SY; *N* = 33), Activity Limitation (AL; *N* = 23), Emotional (EM; *N* = 47), Concerns (CN; *N* = 48), Diabetes Management (DMn; *N* = 63), Diet (DT; *N* = 18) and Work (WK; *N* = 24). Items were rated on a 5-point Likert-type scale with a non-applicable option available when required (Additional Files 1 and 2). Higher scores indicate better QoL outcomes for all domains.

### Sample size

The sample size for the current study was guided by Rasch measurement principles, where stability of item calibration depends on the number of observations rather than traditional power calculations. For scales with more than 20 items, a sample size of 250 is considered sufficient to ensure robust Rasch model estimation, including stable item calibrations [22]. As such, our final sample size of 298 patients exceeded the minimum sample size requirement.

### Psychometric evaluation of the IBs

Our psychometric analysis of the IBs followed the same methodology as related studies for QoL IB development in glaucoma [23, 24], age-related macular degeneration [25], diabetic eye disease [26], and myopia interventions [27]. In brief, Rasch analysis was used to examine the psychometric properties of each IB using Winsteps software (version 5.10.2.0; Chicago, IL, USA) using the Andrich single rating scale model [28]. ‘Not applicable’ responses were treated as structurally missing and did not contribute to threshold or item parameter estimation. Rasch analysis transforms ordinal data into estimates of interval-level data, expressed in log of the odds units (logits) [29]. Rasch analysis also enables the psychometric properties of a scale to be optimized via various ‘fit statistics’. The ‘fit statistics’ used in this study are described briefly below (see Additional Files 1 and 3–9 for detailed information). Analytic performance criteria were used by the study team (Additional File 1) to guide decision making regarding keeping or removing domains and items. For domains, the criteria included alignment with key fit statistics (e.g. unidimensionality), applicability, ceiling effects, test information function, item separation index, measurement range, and clinical importance. For items, criteria included item fit, differential item functioning (DIF; item bias), applicability, discrimination, and clinician and patient importance rating. Following psychometric evaluation, item locations (i.e. item ‘difficulty’ ratings) and thresholds (i.e. step calibrations between each response option) for the CAT algorithm were exported from Winsteps software to build the CAT algorithm.

#### Rating scale assessment

Generally, we expect to see a higher probability of patients with lower ‘ability’ levels of the measured construct selecting the more ‘difficult’ response options for each item. This is evaluated by viewing the category probability curve graphs; a distinct peak for each response option is expected (Additional Files 10–17) [30].

#### Precision

Measurement precision refers to how sensitive the IBs are in distinguishing between patients with high and low levels of the measured construct. A person separation index (PSI) and person reliability (PR) > 2.0 and > 0.8, respectively, are expected [29]. Extreme minimum or maximum scores were removed from the analysis during the optimization process as they were not informative for establishing item measures [29].

#### Unidimensionality

For unidimensional measurement, each IB should measure a single latent construct as multidimensionality makes score interpretation challenging. Principal components analysis of residuals (PCA) was primarily used to assess unidimensionality [31]. Multidimensionality was considered present if the eigenvalue of the first contrast was ≥ 3 and the raw variance explained by measures was < 50%. In addition, four additional indices, including misfitting item groups, standardized residual loadings > 0.4, ratio of raw variance explained by the main dimension and the unexplained variance of a potential secondary dimension, and the disattenuated correlations between the main dimension and other potential dimensions, were considered (Additional File 1) [31].

#### Item fit statistics

‘Item fit’ indicates if patients are responding as expected based on the item’s difficulty and their own ‘ability’ level. Infit and outfit MnSq statistics were assessed to determine item fit (acceptable range: 0.5–1.5) [29]. In the case of misfit, the z-residuals of individual patient responses was evaluated to diagnose potential issues (Additional File 1) [29]. If misfit could not be resolved, item deletion was considered.

#### Local item dependency (LID)

LID occurs when the response to one item is influenced by the response to another item. Ideally, there should be no correlation between the residuals of two items after the effect of the underlying construct is accounted for [32]. We considered LID between two items to be present if the correlation of residuals was > 0.2.

#### Targeting

Targeting describes how well the difficulty level of items targets patients’ ability level. Two metrics were suggestive of poor targeting: (1) gaps in item coverage, as assessed visually using the person-item (Wright) map (Additional Files 10–17); and (2) a difference of > 1.0 logit [33, 34]s between the mean item difficulty and person ability [29].

#### Differential item functioning

DIF indicates if certain items are answered differently (i.e. bias) for particular sample sub-groups (e.g. men vs. women) [34]. DIF was assessed using the iterative-logit (Rasch-Welch) method, and a DIF contrast of > 1.0 logits with an associated Rasch-Welch probability of *P* < 0.05 indicated substantial DIF [33, 34]. Bonferroni correction was applied for multiple DIF tests (p-value / no. items). We examined uniform DIF for age group (≤ 55 vs. >55 years), gender, language of interview (English vs. Mandarin/Malay), DM duration (≤ 13 vs. >13 years), no. DM complications (0–1 vs. >1), and ethnicity (Chinese, Malay, Indian).

#### Measurement range

A larger measurement range demonstrates that an IB can measure a greater spectrum of the associated latent construct. The difference in logits between the ‘easiest’ and ‘hardest’ items was used to calculate measurement range.

#### Test Information Function (TIF)

The TIF provides the sum of information provided by all IB items and identifies where the test has highest/lowest SE. A TIF of ≥ 10 is generally considered excellent [35], and a higher level of test information indicates greater measurement precision (i.e. low SE) at that point on the scale [36].

#### Item separation index (ISI)

Item separation provides information on construct validity. Low item separation (< 3.00) suggests a low difficulty range between items and/or that the person sample is too small to confirm the item difficulty hierarchy [37].

#### Level of dependence between different IBs

To demonstrate that each IB was measuring an independent construct (Pearson’s correlation coefficient of *r* < 0.8), we assessed the correlations between individual person measures from the seven final IBs [38].

### CAT simulations

Following Rasch analyses, the item structure calibrations were exported. We performed simulations to assess the efficiency of our threshold calibrations (JMLE; Joint Maximum Likelihood Estimation method) and associated CAT algorithm [39] in 1000 simulated respondents using R Statistical Computing Environment [40] (“catR” package [41]). Simulations were based on a standard normal distribution (M = 0, SD = 1) and used the Rating Scale Model (RSM), the ML (maximum likelihood) estimator and the Maximum Fisher Information (MFI) item selection criteria [42]. The mean number of items needed was determined for two stopping rules: (1) “high precision” (SE = 0.30; reliability approx. 0.91); and (2) “moderate precision” (SE = 0.387; reliability approx. 0.85). A third, combination stopping rule (SE = 0.3 and maximum test length = 10 items) was used to demonstrate mean measurement precision attained [38]. The accuracy of ability estimation by the CAT algorithm relative to the true simulated values was estimated using the root mean square error (RMSE) and level of bias. Low values indicate that the CAT procedure recovers the underlying trait levels with minimal systematic error and variability under model-congruent conditions (i.e. the data-generating model (RSM) is the same as the model used for estimation). Correlations between simulated person measure estimates from the IBs and CAT were assessed using Pearson’s correlation coefficient. We hypothesized high (*r* ≥ 0.85) and moderate-high (0.75 ≥ *r* < 0.85) correlations for the high and moderate precision stopping rules, respectively. Overall results are summarized in Tables 3 and 4, and results per decile are summarized in Additional Files 18–24. Each decile included 100 simulated respondents, with D1 and D10 representing simulated respondents at the lowest and highest ‘ability’ level, respectively, across the latent trait.

## Results

### Sociodemographic and clinical characteristics

Of the 298 patients (mean (SD) age 60.7 (10.8) years; 57.4% male; duration of DM 15.3 (9.7) years), over half (*n* = 158, 53.0%) managed their DM with diet/exercise and medication, 52 (17.4%) used diet/exercise, medication and insulin, 48 (16.1%) used medication only, and 36 (12.1%) used medication and insulin injections (all self-reported; Table 1). Patients had a mean (SD) HbA1c of 7.9% (1.4%), with 146 (49.0%), 44 (14.8%), 52 (17.5%) and 59 (19.8%) reporting having diabetic retinopathy, diabetic nephropathy, foot complications/neuropathy, and erectile dysfunction, respectively. Just over 40% (*n* = 121) of patients reported having none/a little support with their DM, while 93 (32.2%) and 84 (28.2%) reported having a moderate/great deal of support, respectively.

**Table 1:** Sociodemographic and clinical characteristics of the 298 patients

Variable	*N*	%^a^
*Gender*		
Male	171	57.4%
*Ethnicity*		
Chinese	213	71.5%
Malay	36	12.1%
Indian	49	16.4%
*Marital status*		
Single	49	16.4%
Married	215	72.1%
Divorced/separated/widowed	34	11.4%
*Employment status*		
Working	183	61.4%
Not working	115	38.6%
*Education*		
None or primary school	37	12.4%
Secondary level	112	37.6%
A level,	11	3.7%
Polytechnic diploma, vocational training	52	17.4%
Undergraduate or postgraduate university degree	86	28.9%
*Monthly household income ($SGD)*		
Under $2000	68	22.8%
$2000 to less than $5000	81	27.2%
$5000 to less than $10,000	70	23.5%
$10,000 or more	46	15.4%
*Housing type*		
HDB 1–2 rooms	23	7.7%
HDB 3–4 rooms	135	45.3%
HDB 5 rooms/executive flat	84	28.2%
Condo/landed property	56	18.8%
*Chronic health conditions (self-reported)*		
Hypertension	179	57.4%
High cholesterol	218	73.2%
Heart disease^b^	33	11.1%
Stroke	21	7.0%
Peripheral artery disease	6	2.0%
Depression	9	3.0%
Others	118	39.6%
*DM treatments (self-reported)*		
Diet/exercise only	4	1.3%
Medication only	48	16.1%
Diet/exercise and medication	158	53.0%
Diet/exercise, medication & insulin injections	52	17.4%
Medication and insulin injections	36	12.1%
*DM complications (self-reported)*		
Diabetic retinopathy	146	49.0%
Diabetic nephropathy	44	14.8%
Foot complications	38	12.8%
Neuropathy	14	4.7%
Impotency	59	19.8%
*First-degree relative with DM*		
Yes	230	77.2%
*Amount of support for DM*		
None/A little	121	40.6%
Moderate	93	32.2%
A great deal	84	28.2%
*Satisfaction with level of DM support*		
Not satisfied/Somewhat satisfied	123	41.3%
Very satisfied	146	49.0%
Extremely satisfied	28	9.4%
Continuous variables	Mean/Median	SD/IQR
Age (years)	60.7/63.0	10.8/14.0
Duration of DM (years)	15.3/14.5	9.7/14.5
HbA1c (%)	7.9%/7.6%	1.4%/1.8%
Total cholesterol	4.1/4.0	1.0/1.2
HDL	1.3/0.3	1.3/0.4
LDL	2.1/2.0	0.9/1.0
TG	1.7/1.3	1.3/1.1
BMI	26.3/25.5	4.8/6.4
SBP	139.6/139.0	19.0/24.5
DBP	74.7/74.7	9.0/11.7

^a^Percentages for some variables may not equal 100% due to missing data or patients selecting > 1 category

^b^Includes heart failure, heart attack

BMI, body mass index; DBP, diastolic blood pressure; DM, diabetes mellitus; HDB, housing development board; HDL, high density lipoprotein; IQR, interquartile range; LDL, low density lipoprotein; SD, Standard deviation; SGD, singapore dollars; SBP, systolic blood pressure; TG, triglycerides

### Psychometric properties of the IBs

**Fig. 1:**
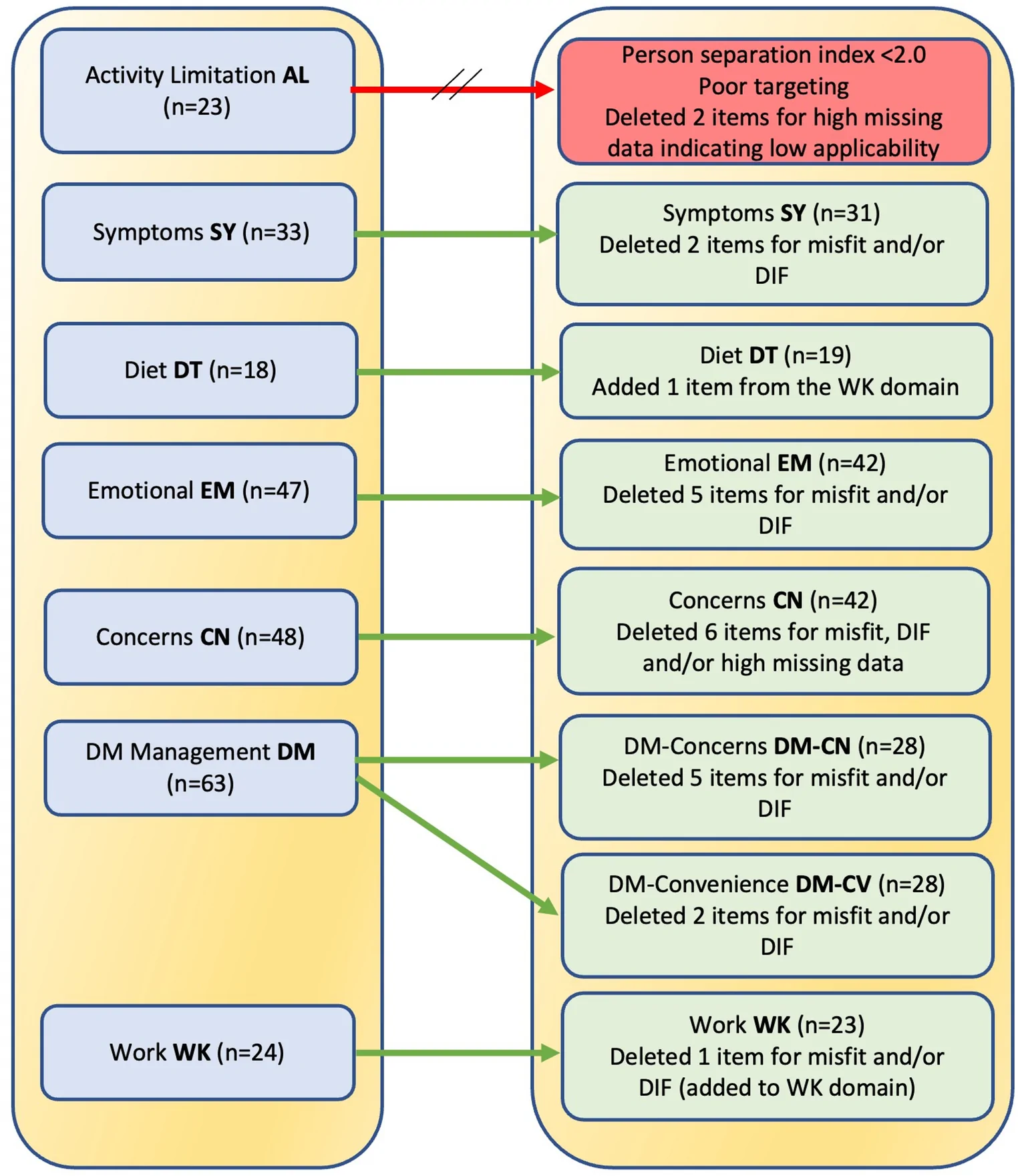
Flowchart depicting the final outcomes for each IB following psychometric modification. On the lefthand side, the seven original item banks are represented, of which six were retained (right, green boxes) and one was not further pursued in CAT testing due to unresolvable psychometric properties (right, red box)

After Rasch-guided modifications (Fig. 1 and Additional Files 3–9), there were seven IBs comprising 213 items: SY (*n* = 31); DT (*n* = 19); EM (*n* = 42); CN (*n* = 42); DMn-Concerns (DMn-CN; *n* = 28); DMn-Convenience (DMn-CV; *n* = 28); and WK (*n* = 23). In brief, SY, DT, WK, EM, and CN displayed acceptable measurement performance overall after deleting items for unresolvable misfit and/or DIF (range 1 item [WK]—6 items [CN]; Fig. 1). The WK item deleted (“Not being able to find diabetes-friendly food at work”) was successfully rehomed in the DT IB. Due to their greater number of psychometric issues, more details are provided for the AL and DM domains below.

#### Activity limitation

The AL IB required deletion of two items for very high missing data (~ 50% for both) indicating low applicability. Despite this amendment, AL did not reach adequate precision levels (PSI = 1.71) and targeting was poor (difference between person and item means > 3.0 logits; Additional File 3). This, combined with a low clinical importance rating by the study team, led to the decision not to pursue this domain further.

#### DM management

The DMn IB initially displayed evidence of multidimensionality, namely PCA variance by the 1st factor < 50%, 1st contrast eigenvalue 5.8, six misfitting items, and standardized residual item loadings relating to ‘convenience’ >0.4 (Additional File 8). Consequently, the DM IB was split into two independent scales capturing items relating to ‘Convenience’ (DMn-CV; *N* = 30) and ‘Concerns’ (DMn-CN; N = 33) and separate Rasch analyses were conducted on each.

The DMn-CV IB had some indicators of multidimensionality (variance by 1st factor 47.3%, 1st contrast eigenvalue 3.3, and three misfitting items), and DIF for gender for one item (Additional File 8). Upon deletion of two items for misfit and/or DIF, precision and measurement range increased, and targeting improved. While the multidimensionality indicators persisted, they were close to the desired cut-off values, and inspection of potential secondary dimensions revealed that they were likely related measurement ‘strands’ rather than separate constructs.

The DMn-CN IB initially had good precision and unidimensionality; however, multiple items demonstrated misfit and/or DIF (Additional File 8). Upon removal of five items for misfit and/or DIF, the scale displayed good measurement precision, unidimensionlity and was free from DIF. While three items still displayed misfit, they were retained as infit/outfit MnSq values were close to desired cut-off values and they were deemed clinically important.

#### Targeting and item coverage

Targeting was suboptimal for all IBs with items, on average, too ‘easy’ for patients (difference in person and item means ranging from 1.30 for DMn-CN to 3.66 for EM; Additional Files 10–17). Inspection of the person-item maps revealed a lack of ‘difficult’ items to target the ‘ability’ level of many of the patients in the sample (Additional Files 10–17). This observed targeting pattern indicates that item thresholds are primarily concentrated at lower levels of the latent trait, resulting in reduced measurement precision at the higher end. Importantly, this limitation is more pronounced on the “healthy”/less impaired side of the scale, reflecting the relatively high-functioning study sample. Inspection of category probability curves indicated that most item thresholds were located between − 2.0 and 2.0 logits, consistent with the peak in test information at θ ≈ 0 to 1 logits.

#### Level of dependence between different IBs

Correlation coefficients of the person measures for the final IBs supported the independence of each QoL domain (*r* < 0.8, range: 0.47–0.71; Table 2).

**Table 2:** Correlation coefficients between the final seven domains of the DiabCAT item banks

	SY	DT	EM	CN	DMn-CN	DMn-CV	WK
SY	–	–	–	–	–	–	–
DT	0.50	–	–	–	–	–	–
EM	0.67	0.61	–	–	–	–	–
CN	0.61	0.63	0.71	–	–	–	–
DM-CN	0.49	0.64	0.61	0.75	–	–	–
DM-CV	0.47	0.67	0.55	0.46	0.54	–	–
WK	0.51	0.53	0.62	0.61	0.57	0.56	–

CN, concerns; DMn-CN, DM management-concerns; DMn-CV, DM management-convenience; DT, diet; EM: emotional; SY, symptoms; WK, work

### CAT simulations

Low RMSE and bias values indicated that the CAT algorithm accurately recovered the underlying trait levels under model-congruent conditions, with minimal systematic error and overall estimation error. The mean number of items administered for the ‘high precision’ 0.3 SE stopping rule ranged between 13.9 for DMn-CV and 18.3 for EM (overall mean 15.1 items; Table 3). Compared to using all available items in the IBs, using CAT resulted in percentage reductions ranging from 24.2% (DT) to 65.2% (CN). The target stopping rule was met ≥ 95% for most IBs across moderate ability levels (i.e. ~D4-D9) with lower proportions at the very ‘unable’ (D1) and very ‘able’ (D10) ends of the spectrum (Additional Files 18–24).

For the ‘moderate’ precision stopping rule (0.387 SE), the mean number of items administered ranged between 8.7 for DMn-CV and 11.2 for EM (overall mean 9.6 items; Table 3), with percentage reductions ranging from 48.4% (DT) to 78.8% (CN). Except for the extreme deciles (D1 and D10), the proportion of simulated respondents reaching the stopping rule target was > 80% (Additional Files 18–24).

Correlations between the CAT simulated person measures and the IBs were moderate to high (range: 0.76–0.81 and 0.76–0.79 for high and moderate precision levels, respectively; Table 3).

In simulations with the combined 10-item/SE stopping rule, the SE achieved ranged between 0.342 for CN and 0.412 for MB (reliability ~ 0.88 and ~ 0.83, respectively (Table 4)).

**Table 3:** CAT simulation results for the seven DiabCAT quality of life item banks

Domain, items	Mean no. items	Correlation with true theta	RMSE	Bias	% satisfied stopping rule
*SE 0.3 (high precision)*					
SY, 31	15.1	0.79	0.82	− 0.15	92.4%
DT, 19	14.4	0.77	1.00	− 0.05	75.0%
EM, 42	18.3	0.76	0.92	0.07	97.2%
CN, 42	14.6	0.79	0.78	0.03	96.5%
DMn-CV, 28	13.9	0.80	0.77	− 0.06	91.3%
DMn-CN, 28	14.5	0.77	0.92	0.01	86.1%
WK, 23	14.8	0.81	0.77	− 0.08	84.4%
*SE 0.387 (moderate precision)*					
SY, 31	9.5	0.78	0.85	− 0.15	95.2%
DT, 19	9.8	0.77	1.00	− 0.01	85.7%
EM, 42	11.2	0.76	0.93	0.08	100%
CN, 42	8.9	0.78	0.81	0.07	98.9%
DMn-CV, 28	8.7	0.79	0.81	− 0.07	96.7%
DMn-CN, 28	9.8	0.76	0.94	0.04	91.4%
WK, 23	9.4	0.79	0.82	− 0.10	93.8%

CAT, computerized adaptive test; CN, concerns; DMn-CN, diabetes management-concerns; DMn-CV, diabetes management-convenience; DT, diet; EM, emotional; SY, symptoms; WK, work; RMSE, root mean square error; SE, standard error of measurement

**Table 4:** CAT simulation results using a combined stopping rule based on test length and precision^a^

Domain	Mean SE	Reliability^b^	Correlation with true theta	RMSE	Bias
SY, 31	0.365	0.87	0.77	0.91	− 0.16
DT, 19	0.412	0.83	0.75	1.01	0.02
EM, 42	0.391	0.85	0.77	0.94	0.10
CN, 42	0.342	0.88	0.78	0.80	0.05
DMn-CV, 28	0.353	0.88	0.78	0.87	− 0.06
DMn-CN, 28	0.368	0.87	0.75	0.97	0.02
WK, 23	0.363	0.87	0.79	0.83	− 0.10

CAT, computerized adaptive test; CN, concerns; DMn-CN, diabetes management-concerns; DMn-CV, diabetes management-convenience; DT, diet; EM, emotional; SY, symptoms; WK, work; RMSE, root mean square error; SE, standard error of measurement

^a^Test length = 10 items and precision = SE 0.3; ^b^Derived from the formula 1-SE^2^

## Discussion

Our Rasch-guided psychometric assessment of seven T2DM-specific QoL IBs (“DiabCAT”) demonstrated acceptable measurement properties for SY, DT, EM, CN and WK following removal of 15 poorly fitting or biased items, with some limitations in targeting at higher levels of the latent trait. The AL IB, in contrast, displayed unresolvable psychometric issues and was not pursued further. The DMn IB was multidimensional and was split into two unidimensional scales (DMn-CV and DMn-CN). In CAT simulations of the seven final IBs (*N* = 213 items in total), the mean number of items required per IB ranged between 9 (CN, DMn-CV) and 11 (EM) for moderate precision, and 14 (DMn-CV, DT) and 18 (EM) for high precision measurement. Overall, DiabCAT provides researchers and clinicians with a comprehensive new tool to understand the impact of T2DM and associated treatments on QoL with low administration burden, particularly when a ‘moderate’ precision stopping rule is employed.

Despite amendments, our AL IB had suboptimal precision and poor targeting. This finding was unexpected, as DM has long been associated with high physical disability [43], and reduced physical functioning was mentioned by patients during our qualitative phase [20]. Although clinically important, the study team (psychometricians EF, RM, EL and clinicians BYM and EST) opted not to generate calibrations for CAT for AL due to its poor psychometric properties. Alternative measures of generic physical function, such as the SF-36 and the PROMIS physical functioning domain, which have been validated and/or used clinically in people with DM [19], may be good substitutes for measurement of physical function.

Our DMn IB initially displayed strong markers of multidimensionality, suggestive of the presence of a secondary dimension. Multidimensional assessments are problematic because they incorrectly combine different trait proficiencies into a single score, leading to a distorted view of a person’s abilities and reducing measurement quality [31]. In such a large IB (*N* = 63 items) covering a complex topic like DM management, the presence of multidimensionality is perhaps unsurprising. Inspection of the item loadings revealed that items about DM management ‘concerns’ and ‘inconvenience’ formed separate groups and the study team split the DMn scale into two, unidimensional scales measuring discrete aspects of DM management.

Despite the high rate of comorbidities reported by our patients and the relatively poor level of DM control (mean HbA1c 7.9%), items were generally too “easy” for our patients’ average level of ability, particularly for EM. This observed targeting pattern likely reflects the relatively high functioning of the study sample rather than a deficiency in item content. Poor targeting may arise because participants in qualitative studies are typically asked to elaborate on challenges relating to their health condition in order to generate item content, and this may disproportionately emphasize the QoL impact on patients. When multiple items are administered via a questionnaire to a broader population, the effects may become diluted, resulting in ceiling effects as observed in this and related studies [27]. Due to the poor targeting, our CAT efficiency was reduced for the highest deciles (D9-10), where more items were needed to obtain a score estimate. However, as poorly rather than highly functioning patients tend to be the focus of clinician monitoring in healthcare or pharmaceutical drug development targets, this may not be problematic. Future work will consider implementing a predicted standard error reduction (PSER) stopping rule, which stops the CAT when the remaining items are not likely to substantially improve measurement precision [44].

Our moderate precision simulations showed that CAT administration of our DiabCAT IBs could produce seven valid, DM-specific outcomes in < 70 items in total. This compares favorably with other commonly used DM-specific PROMs; for example, many DM-specific questionnaires comprise ~ 20–35 items to measure a single construct such as symptoms (e.g. the Diabetes Symptom Checklist-Revised [16]) or diabetes distress (PAID [9]), while others such as the Asian Diabetes Quality of Life [45] and the ADDQoL [10] comprise multiple domains but with very few (i.e. 2–6) representative items, likely limiting measurement validity due to item insufficiency.

The high-precision stopping rule resulted in lower efficiency for DiabCAT compared with other CAT systems - such as certain PROMIS CATs, which typically require only 4–8 items per IB [46, 47]. This likely reflects differences in CAT design parameters (e.g. stopping rules) and item bank targeting. In particular, reduced coverage at higher trait levels resulted in longer CATs for individuals in this range. Despite this, the overall administration time remains practical. Each DiabCAT domain is expected to take less than three minutes to complete [48, 49], allowing all seven domains to be assessed in approximately 20 min. This enables a comprehensive yet time-efficient evaluation of DM-specific QoL. Importantly, administration burden can be further reduced by limiting the maximum test length, with only minimal loss of precision, as demonstrated by the combination stopping rule. In addition, because each CAT domain operates independently, researchers or clinicians can selectively administer only those domains relevant to their specific outcomes, further improving efficiency. However, correlations between CAT-derived and full IB person measures were slightly lower than anticipated, suggesting a potential trade-off between reducing response burden and maintaining optimal measurement accuracy. This consideration may be particularly relevant in contexts requiring precise individual-level comparisons, where higher reliability is essential. Future studies will therefore evaluate the agreement between CAT and full IB scores in real-world clinical populations to further establish the accuracy of DiabCAT measurements.

DiabCAT provides clinicians with seven independent QoL CATs with which to monitor T2DM management effectiveness from the patient’s perspective and guide shared-decision making, aligning closely with value-based care initiatives [50]. The CATs operate on a web-based application and will be made available in the future via a licensing agreement. Our group has already demonstrated that CAT administration in ophthalmic settings is acceptable, feasible, and time-efficient [51, 52], and work is ongoing to scale the implementation across tertiary eye clinics in Singapore using an implementation science approach.

Our rationale for developing a new PROM for T2DM was based on the premise that current generic PROMs (e.g., PROMIS CATs) may not be sensitive to DM-specific issues; however, we did not test this empirically through content validity studies in patients with DM. Introducing new measures limits comparability of scores between PROMs and introduces complexity in implementing PROMs in clinical practice. Given that several of DiabCAT’s domains measure similar constructs to existing PROMIS ones, future work could focus on developing crosswalks between DiabCAT Symptoms and PROMIS Pain Interference, DiabCAT Emotional and PROMIS Depression, and DiabCAT Concerns with PROMIS Anxiety scores to provide clinical users with a useful tool for comparing scores generated from related instruments.

Strengths of our study include the comprehensive psychometric assessment of the DiabCAT IBs and our systematic, data-driven and stakeholder-guided approach to content modification. Our recruitment of patients across various clinical and demographic groups enhanced the applicability of our calibrations. However, we recruited comparatively fewer Indians and Malays, which may mean our calibrations are less applicable to these groups. Further studies to test the agreement in item calibrations between our Singaporean population and patients with T2DM from other Asian countries are warranted. Regarding our simulations, we used the ML estimator for our CAT simulations, which may have produced inaccurate score estimates at the extremes compared to EAP (expected a posteriori) or BM (Bayesian Method). We also observed a relatively low proportion of responses in the ‘hardest to endorse’ category for each domain (< 5%), which may have reduced the precision of the corresponding threshold estimates. Moreover, given that our item bank calibrations were derived from a population with relatively high mean person measures, our use of the standard normal distribution (M = 0, SD = 1) to run our simulations may have affected the accuracy of our results. Finally, responses were simulated rather than generated from the empirical study sample; therefore, while they provide insight into CAT estimation accuracy under model-congruent conditions, they may not fully reflect measurement precision across the distribution of trait levels observed in clinical practice.

## Conclusions

Following psychometric evaluation, seven DiabCAT IBs comprising 213 items demonstrated good measurement properties notwithstanding suboptimal targeting. Due to psychometric shortcomings, the AL IB was not further developed. CAT simulations with a moderate precision stopping rule revealed that CAT application could reduce the number of items needed by 48–79% (domain dependent) compared to the full IB, producing seven valid, DM-specific outcomes in < 70 items. DiabCAT provides researchers and clinicians a means to obtain a comprehensive yet low-burden understanding of the QoL impact of T2DM and its management. Future work aims to assess the efficiency, validity, and reliability of implementing DiabCAT in real-world clinical care for patients with T2DM.

## Supplementary Information

Below is the link to the electronic supplementary material.

Supplementary Material 1

## Data Availability

The datasets generated and/or analysed during the current study are not publicly available due to future commercialization intent, but are available from the corresponding author on reasonable request.
