# Supplementary material for: Quality of life item banks for type 2 diabetes: psychometric evaluation and computerized adaptive testing simulations
Source: Qual Life Res. 2026 Sep 21;35(10):290. doi: 10.1007/s11136-026-04387-6 (PMC13593726; doi:10.1007/s11136-026-04387-6)
Supplement: Supplementary file 1 [file 11136_2026_4387_MOESM1_ESM.docx]

**Table of Contents**

| Page 2 | **Additional File 1:** Materials and Methods |
| --- | --- |
| Page 5 | **Additional File 2:** Description of seven type 2 diabetes quality of life domains before Rasch analysis |
| Page 7 | **Additional File 3:** Psychometric properties of the Activity limitation domain |
| Page 8 | **Additional File 4:** Psychometric properties of the Symptoms domain |
| Page 9 | **Additional File 5:** Psychometric properties of the Diet domain |
| Page 11 | **Additional File 6:** Psychometric properties of the Emotional domain |
| Page 13 | **Additional File 7:** Psychometric properties of the Concerns domain |
| Page 15 | **Additional File 8:** Psychometric properties of the DM management domain |
| Page 18 | **Additional File 9:** Psychometric properties of the Work domain |
| Page 20 | **Additional File 10:** Category Probability Curves, Test Information Function, and Person-Item Map of the Activity Limitation domain |
| Page 23 | **Additional File 11:** Category Probability Curves, Test Information Function, and Person-Item Map of the Symptoms domain |
| Page 25 | **Additional File 12:** Category Probability Curves, Test Information Function, and Person-Item Map of the Diet domain |
| Page 28 | **Additional File 13:** Category Probability Curves, Test Information Function, and Person-Item Map of the Emotional domain |
| Page 31 | **Additional File 14:** Category Probability Curves, Test Information Function, and Person-Item Map of the Concerns domain |
| Page 34 | **Additional File 15:** Category Probability Curves, Test Information Function, and Person-Item Map of the DMn-Concerns domain |
| Page 36 | **Additional File 16:** Category Probability Curves, Test Information Function, and Person-Item Map of the DMn-Convenience domain |
| Page 38 | **Additional File 17:** Category Probability Curves, Test Information Function, and Person-Item Map of the Work domain |
| Page 41 | **Additional File 18:** CAT simulation results for the Symptoms domain at two precision levels |
| Page 42 | **Additional File 19:** CAT simulation results for the Diet domain at two precision levels |
| Page 43 | **Additional File 20:** CAT simulation results for the Emotional domain at two precision levels |
| Page 44 | **Additional File 21:** CAT simulation results for the Concerns domain at two precision levels |
| Page 45 | **Additional File 22:** CAT simulation results for the DMn-Convenience domain at two precision levels |
| Page 46 | **Additional File 23:** CAT simulation results for the DMn-Concerns domain at two precision levels |
| Page 47 | **Additional File 24:** CAT simulation results for the Work domain at two precision levels |

Quality Of Life Item Banks For Type 2 Diabetes: Psychometric Evaluation and Computerized Adaptive Testing Simulations, Quality of Life Research, Eva K. Fenwick; Ryan Eyn Kidd Man; Aricia Xin Yi Ho; Ester PX Lee; E Shyong Tai; Ngiap Chuan Tan; Sue-Anne Ee Shiow Toh ; Tat Yean Tham; Chin Meng Khoo; Yong Mong Bee; Ecosse L. Lamoureux; Singapore Eye Research Institute and Singapore National Eye Centre, Singapore; [ecosse.lamoureux@duke-nus.edu.sg](mailto:ecosse.lamoureux@duke-nus.edu.sg).

**Additional File 1: Materials and Methods**

*Assessment of T2DM*

T2DM was physician-diagnosed according to 2014 Singapore Medical Council guidelines, i.e. fasting plasma glucose (FPG-no caloric intake for at least 8 hours) >7.0mmol/L, or 2-hour post challenge (oral glucose tolerance test) plasma glucose >11.1mmol/L or casual plasma glucose >11.1 mmol/L.[1]

*Development of the DiabCAT IBs*

A total of 256 items within seven domains were generated from a comprehensive literature review, qualitative sessions with T2DM patients, and feedback from experts, including endocrinologists, diabetic nurses and related allied health professionals. Subsequent item refinement was performed via expert panel sessions and pre-tested in T2DM patients.[2] Items in all domains apart from DM Management and Diet were preceded by the statement “*Because of your diabetes and its management…”* and rated on 5-category scales, with a 1 month timeframe specified for the Emotional (EM), Concerns (CN) and Symptoms (SY) domains.

*Analytic performance criteria guiding decision making*

| **Analytic performance criteria for determining domain and item viability** | |
| --- | --- |
| **Parameter** | **Desirable values** |
| *Domain* | |
| Model fit statistics (i.e. ordered thresholds, unidimensionality, precision level) | Present |
| Applicability | >80% response rate (% of participants providing answers to >75% items in the domain) |
| Ceiling effect | <20% ceiling effect (i.e. extreme high responders) |
| Test Information Function (TIF) | ≥10 |
| Item Separation Index (ISI) | ≥3.00 |
| Measurement range | >2.0 logits |
| Clinical importance | High (as determined by the psychometrician and clinical teams) |
| *Item* | |
| **Parameter** | **Desirable values** |
| Item fit | Infit/outfit MnSq <1.5*  *If ≥1.5, able to be reduced to <1.5 by removal of responses with high z-residuals based on the process outlined by Boone and colleagues.[3] |
| Differential Item Functioning (DIF) | None present |
| Applicability | <25% missing data |
| Discrimination* | Not substantially below 1.0 |
| Clinical importance | High (as determined by the psychometrician and clinical teams) |
| Patient importance rating | High (Frequently mentioned during focus group or interview discussions and/or deemed important during consultations post-psychometric testing) |
| *Although Rasch models assume equal discrimination across items, empirical item discriminations never are exactly equal. Winsteps software reports an estimate of those discriminations post-hoc (as a type of fit statistic). The amount of the departure of a discrimination from 1.0 is an indication of the degree to which that item misfits the Rasch model. | |

*2.3.1 Rating Scale Assessment*

Participants should be more likely to choose the ‘harder’ response categories (i.e. the average observed measures should increase monotonically) as person ‘ability’ decreases. If response category disordering is detected, it may suggest underused, unclear, or excess response categories.[4, 5]

*2.3.2 Precision*

High PSI and PR values mean that there is a high probability that persons estimated to have high scores actually do have higher scores than persons estimated with low scores (i.e. indicating better discrimination of the IBs for the current sample). In contrast, low PSI and PR values suggest that the IBs may not have sufficient sensitivity to distinguish between high and low performers.

*2.3.3 Unidimensionality*

We assessed four additional unidimensionality metrics, including:

1. Presence of groups of misfitting items, potentially indicating poor fit to the underlying construct

2. Standardized residual loadings of items >0.4 potentially indicating a secondary dimension

3. The ratio of the raw variance explained by the main ‘Rasch’ dimension and the unexplained variance of a potential secondary dimension. A higher ratio is suggestive of unidimensionality (i.e., the Rasch dimension is noticeably larger)

4. Disattenuated correlations between the Rasch dimension and other potential dimensions. High disattenuated correlations (≥0.82) are indicative of unidimensionality and support the likelihood that a potential secondary dimension is a “strand” of the main construct rather than a separate construct.[6]

If multiple indicators suggested multidimensionality, splitting the scale into >1 unidimensional constructs was considered.

*2.3.4 Item Fit Statistics*

Z-residuals of > 4 and < -4 were considered to indicate an erroneous/unpredictable response. Such responses were given a weightage of zero and item fit statistics were iteratively reevaluated. In the case of persistent misfit, the process was repeated for z-residuals >|3| and then >|2|, until satisfactory item fit statistics were obtained.[3] This iterative process was applied to minimise the undue influence of a small number of highly unexpected responses on item calibration that likely reflect measurement artefacts rather than meaningful variation in the construct of interest, while retaining the full sample in the analysis.

Item discrimination was also important in assessing item performance. Over-discriminating items (>1.0) tend to discriminate between high and low performers more than expected, while for under-discriminating items (<1.0) the opposite is true.[7] Given that under-discrimination is more damaging to measurement, items with values substantially under 1.0 were considered for deletion.

*2.3.5 Local Item Dependency (LID)*

We used a specific process of anchoring LID-free person measures to all other person measures in each IB to produce LID-free item calibrations. This forces item difficulties and rating-scale structures within the IB to conform with the LID-free person measures and prevents LID from impacting item difficulties.[8]

**References**

1.	Goh, S.Y., et al., *Ministry of Health clinical practice guidelines: diabetes mellitus.* Singapore medical journal, 2014. **55**(6): p. 334.

2.	Fenwick, E., et al., *Developing content for a diabetes-specific quality of life item bank and computerized adaptive testing system (DiabCAT).* Advances in Patient-Reported Outcomes, 2026. **2**(2): p. 100329.

3.	Boone, W., J. Staver, and M. Yale, *Rasch Analysis in the Human Sciences*. 2014, Dordrecht: Springer.

4.	Khadka, J., Gothwal, V.K., McAlinden, C., Lamoureux, E.L., Pesudovs, K., *The importance of rating scales in measuring patient-reported outcomes.* Health Qual Life Outcomes, 2012. **10**: p. 80-93.

5.	Linacre, J.M., *Optimizing rating scale category effectiveness.* J Appl Meas, 2002. **3**: p. 85-106.

6.	Schumacker, R. and P. Muchinsky, *Disattenuating correlation coefficients.* Rasch Measurement Transactions, 1996. **10**(1): p. 479.

7.	Linacre, M. *DISCRIMINATION= report item discrimination = No*. 1999; Available from: <https://www.winsteps.com/winman/discrim.htm>.

8.	Fenwick, E.K., et al., *Diabetic Retinopathy and Macular Edema Quality-of-Life Item Banks: Development and Initial Evaluation Using Computerized Adaptive Testing.* Investigative Opthalmology & Visual Science, 2017. **58**(14): p. 6379.

| **Additional File 2:** Description of seven type 2 diabetes quality of life domains before Rasch analysis | | | |
| --- | --- | --- | --- |
| **Quality of life domain** | **Description** | **Item stem** | **Response options** |
| ***Because of your diabetes and its management…*** | | | |
| Activity Limitation | Day to day tasks (e.g. shopping) and physical functioning (e.g. moderate physical activity) | How much difficulty do you have…? | *None, A little, Quite a bit, A lot,* *Unable to do because of my diabetes*  *Non-appliable option: Does not apply to me* |
| Symptoms | Unwanted symptoms arising from diabetes and its management (e.g. dizziness, stomach problems, body aches). | In the past 1 month, how bothered were you by…? | *Not at all, A little bit, Moderately, Very,* *Extremely* |
| Diet | Challenges associated with diet control (e.g. needing to change dietary habits or feeling pressured to eat diabetes unfriendly food in social situations) | How much trouble is…?  How concerned are you about…? | *Not at all, A little bit, A moderate amount, A lot,* *Too much trouble to do*  *Not at all, A little bit, Moderately, Very, Extremely*  *Non-appliable option: Does not apply to me* |
| Emotional | Emotional reactions to having and managing DM (e.g. overwhelm, loss of self-worth) | In the past 1 month, how often did you feel…? | *None of the time, A little of the time, Some of the time, Most of the time, All of the time* |
| Concerns | Concerns about safety, disease progression, lifestyle restrictions; how DM affects personal and social life | In the past 1 month, how concerned were you about…? | *Not at all, A little bit, Moderately, Very,* *Extremely*  *Non-appliable option: Does not apply to me* |
| DM management | Challenges and concerns associated with managing DM (e.g. management burden, controlling DM, taking medications/insulin, and costs) | How much trouble is…?  How concerned are you about…? | *Not at all, A little bit, A moderate amount, A lot,* *Too much trouble to do*  *Not at all, A little bit, Moderately, Very,* *Extremely*  *Non-appliable option: Does not apply to me* |
| Work | Impact of DM on work performance, relationships and opportunities, and financial impact | Currently, how concerned are you about...?  Currently, how much difficulty do you have...? | *Not at all, A little bit, Moderately, Very,* *Extremely*  *None, A little, Quite a bit, A lot,* *Unable to do because of my diabetes*  *Non-appliable option: Does not apply to me* |

| Additional File 3: Psychometric properties of the Activity limitation domain (N=297) | | | |
| --- | --- | --- | --- |
| Parameters | Rasch model | First iteration (n=297)* | Final iteration§ (n=261) |
| Disordered thresholds | No | No | No |
| No. of items | - | 23 | 21 |
| Person separation index | >2.0 | **1.49** | **1.71** |
| Person reliability | >0.8 | **0.69** | **0.75** |
| Item separation index | >3.0 | 6.00 | 6.54 |
| PCA, variance by 1^st^ factor | >50% | **47.7%** | **47.6%** |
| PCA, Eigenvalue for 1^st^ contrast | <3.0 | 2.09 | 2.10 |
| PCA, ratio % raw variance explained by items: % raw variance explained by 1^st^ contrast | Larger ratio = better | 16.7% : 4.9% (3.4) | 17.2% : 5.2% (3.3) |
| Item fit (infit MnSq) | <1.5 | None | None |
| Item fit (outfit MnSq) | <1.5 | None | None |
| DIF‡ | <1.0 logits and p<0.05 | None | None |
| Measurement range | >2.0 logits | 4.22 | 4.22 |
| Missing data | <25% | **AL4 (48.2%), AL17 (49.5%)** | None |
| Targeting, difference between person & item means | <1.0 logits | **3.74** | **3.29** |
| TIF | >10 | 17.0 | 15.6 |
| AL4=Carrying out your household/family responsibilities; AL17=Driving  *Ceiling effect: 36, 12.1%  §Dropped 36 participants with ‘extreme’ high responses; deleted 2 items for high missing data indicating low applicability (AL4, AL17).  ‡DIF was assessed for age group (≤55 vs. >55 years), gender, language of interview (English vs. Mandarin/Malay), DM duration (≤13 vs. >13 years), No. DM complications (0-1 vs. >1), ethnicity (Chinese, Malay, Indian)  Bolded values represent poor fit to the Rasch model.  DIF=Differential item functioning; LID=Local item dependency; PCA=Principal components analysis; TIF=Test information function | | | |

| Additional File 4: Psychometric properties of the Symptoms domain (N=296) | | | |
| --- | --- | --- | --- |
| Parameters | Rasch model | First iteration (n=296)* | Final iteration (n=277)§ |
| Disordered thresholds | No | No | No |
| No. of items | - | 33 | 31 |
| Person separation index | >2.0 | **1.79** | 2.09 |
| Person reliability | >0.8 | **0.76** | 0.81 |
| Item separation index | >3.0 | 4.46 | 4.65 |
| PCA, variance by 1^st^ factor | >50% | **37.0%** | **38.4%** |
| PCA, Eigenvalue for 1^st^ contrast | <3.0 | 2.2 | 2.1 |
| PCA, ratio % raw variance explained by items: % raw variance explained by 1^st^ contrast | Larger ratio = better | 9.3% : 4.2% (2.2) | 8.3% : 4.2% (2.0) |
| Item fit (infit MnSq) | <1.5 | **SY31 (1.62); SY16 (1.57); SY29 (1.60)** | Noneγ |
| Item fit (outfit MnSq) | <1.5 | **SY31 (2.12); SY16 (1.66); SY24 (1.52)** | Noneγ |
| DIF‡ | DIF contrast <1.0 logits and p<0.05 | **Gender: SY16 (1.82)** | None |
| Measurement range | >2.0 logits | 2.14 | 2.28 |
| Missing data | <25% | None | None |
| Targeting, difference between person & item means | <1.0 logits | **2.79** | **2.65** |
| TIF | >10 | 32.1 | 28.9 |
| SY16=Sexual difficulties; SY24=Nausea/Vomiting; SY29=Swelling in your legs or feet; SY31=Unintentional weight loss  *Ceiling effect: 19 (6.4%)  γHigh item misfit for 2 items was resolved by diagnosing misfit through unpredictable individual responses (total n=5) and giving errant responders a weighting of 0 – see methods  §Dropped 19 participants with ‘extreme’ high responses; deleted item SY16 for misfit and DIF, and SY31 for misfit.  ‡DIF was assessed for age group (≤55 vs. >55 years), gender, language of interview (English vs. Mandarin/Malay), DM duration (≤13 vs. >13 years), No. DM complications (0-1 vs. >1), ethnicity (Chinese, Malay, Indian)  LID >0.2 = 14 pairs of 466, 3.0%  Bolded values represent poor fit to the Rasch model.  DIF=Differential item functioning; LID=Local item dependency; PCA=Principal components analysis; TIF=Test information function | | | |

| Additional File 5: Psychometric properties of the Diet domain (N=296) | | | | | |
| --- | --- | --- | --- | --- | --- |
| Parameters | Rasch model | **First iteration (n=296)*** | Final iteration (n=277)§ | Diet+WK19 First iteration* (N=296) | Diet+WK19 Final iteration (N=277)§ |
| Disordered thresholds | No | No | No | No | No |
| No. of items | - | 18 | 18 | 19 | 19 |
| Person separation index | >2.0 | **1.88** | 2.13 | 1.90 | 2.16 |
| Person reliability | >0.8 | **0.78** | 0.82 | 0.78 | 0.82 |
| Item separation index | >3.0 | 4.60 | 4.60 | 4.36 | 4.36 |
| PCA, variance by 1^st^ factor | >50% | 50.3% | 50.3% | 50.2% | 50.2% |
| PCA, Eigenvalue for 1^st^ contrast | <3.0 | 2.2 | 2.2 | 2.2 | 2.2 |
| PCA, ratio % raw variance explained by items: % raw variance explained by 1^st^ contrast | Larger ratio = better | 10.2% : 6.2% (1.6) | 8.6% : 6.2% (1.4) | 9.6% : 5.9% (1.6) | 9.6% : 5.9% (1.6) |
| Item fit (infit MnSq) | <1.5 | **DT8 (1.58)** | Noneγ | **DT8 (1.59)** | Noneγ |
| Item fit (outfit MnSq) | <1.5 | **DT14 (1.78)** | Noneγ | **DT14 (1.82), DT8 (1.51)** | Noneγ |
| DIF‡ | <1.0 logits and p<0.05 | None | None | None | None |
| Measurement range | >2.0 logits | 1.60 | 1.58 | 1.56 | 1.55 |
| Missing data^ | <25% | **DT1, DT2, DT10, DT11, DT17 (28.7%); DT8 (32.1%)** | **As per First iteration** | **As per First iteration + WK19 (41.2%)** | **As per First iteration + WK19 (41.2%)** |
| Targeting, difference between person & item means | <1.0 logits | **1.81** | **1.64** | **1.81** | **1.65** |
| TIF | >10 | 18.1 | 17.5 | 19.3 | 18.5 |
| DT8=Preparing diabetes-friendly food for family and friends; DT14=Being pressured into eating unhealthy food  *Ceiling effect: 19 (6.4%)  γ High item misfit for 2 items was resolved by diagnosing misfit through unpredictable individual responses (total n=7) and giving errant responders a weighting of 0 – see methods  §Dropped 19 participants with ‘extreme’ high responses; added item WK19=Not being able to find diabetes-friendly food at work  ‡DIF was assessed for age group (≤55 vs. >55 years), gender, language of interview (English vs. Mandarin/Malay), DM duration (≤13 vs. >13 years), No. DM complications (0-1 vs. >1), ethnicity (Chinese, Malay, Indian)  ^Most of these items cannot be answered if the patient doesn’t consider themselves to have/use a diet plan. During CAT administration, these items will be not be administered if the patient responds ‘No’ to an initial screening question “Do you use a diet plan to manage your diabetes?”, and therefore they will not contribute to response burden if not applicable.  LID >0.2 = 5 pairs of 171, 2.9%  Bolded values represent poor fit to the Rasch model.  DIF=Differential item functioning; LID=Local item dependency; PCA=Principal components analysis; TIF=Test information function | | | | | |

| Additional File 6: Psychometric properties of the Emotional domain (N=297) | | | |
| --- | --- | --- | --- |
| Parameters | Rasch model | First iteration (n=297) | Final iteration§ (n=232)* |
| Disordered thresholds | No | No | No |
| No. of items | - | 47 | 42 |
| Person separation index | >2.0 | **1.76** | 2.53 |
| Person reliability | >0.8 | **0.76** | 0.86 |
| Item separation index | >3.0 | 4.69 | 4.54 |
| PCA, variance by 1^st^ factor | >50% | **46.6%** | **48.5%**† |
| PCA, Eigenvalue for 1^st^ contrast | <3.0 | **3.1** | 3.0 |
| PCA, ratio % raw variance explained by items: % raw variance explained by 1^st^ contrast | Larger ratio = better | 11.1% : 3.5% (3.2) | 7.7% : 3.7% (2.1) |
| Item fit (infit MnSq) | <1.5 | **EM43 (1.84), EM31 (1.97), EM41 (1.70)** | Noneγ |
| Item fit (outfit MnSq) | <1.5 | **EM43 (4.02), EM31 (2.67), EM41 (1.90), EM19 (1.84), EM45 (1.53), EM16 (1.52)** | Noneγ |
| DIF‡ | <1.0 logits and p<0.05 | **Lang: EM9 (-1.29), EM31 (-1.09), EM18 (-1.16)** | None |
| Measurement range | >2.0 logits | 3.43 | 3.03 |
| Missing data | <25% | None | None |
| Targeting, difference between person & item means | <1.0 logits | **4.00** | **3.66** |
| TIF | >10 | 36.4 | 29.2 |
| EM9=Burnt out (e.g. mentally, physically and/or emotionally exhausted); EM16=Guilty; EM18=Unmotivated to perform your daily duties and responsibilities; EM19=That life is unfair; EM31=Reluctant to socialise; EM41=Like diabetes is always on your mind; EM43=Like you have to hide your diabetes from others  *Ceiling effect: 65 (21.9%)  γHigh item misfit for 5 items was resolved by diagnosing misfit through unpredictable individual responses (total n=15) and giving errant responders a weighting of 0 – see methods  §Deleted 1 item for misfit and DIF for ‘language’ (EM31), 2 items for misfit (EM41, EM43) and 2 items for DIF for ‘language’ (EM9, EM18).  †Disattenuated correlations = 1.0  ‡DIF was assessed for age group (≤55 vs. >55 years), gender, language of interview (English vs. Mandarin/Malay), DM duration (≤13 vs. >13 years), No. DM complications (0-1 vs. >1), ethnicity (Chinese, Malay, Indian)  LID>0.2 = 43 of 861, 5.0%  Bolded values represent poor fit to the Rasch model.  DIF=Differential item functioning; LID=Local item dependency; PCA=Principal components analysis TIF=Test information function | | | |

| Additional File 7: Psychometric properties of the Concerns domain (N=297) | | | |
| --- | --- | --- | --- |
| Parameters | Rasch model | **First iteration (n=297)*** | Final iteration (n=291)§ |
| Disordered thresholds | No | Noφ | Noφ |
| No. of items | - | 48 | 42 |
| Person separation index | >2.0 | 3.20 | 3.68 |
| Person reliability | >0.8 | 0.91 | 0.93 |
| Item separation index | >3.0 | 6.80 | 8.16 |
| PCA, variance by 1^st^ factor | >50% | 54.4% | 56.8% |
| PCA, Eigenvalue for 1^st^ contrast | <3.0 | 3.7† | **3.1** |
| PCA, ratio % raw variance explained by items: % raw variance explained by 1^st^ contrast | Larger ratio = better | 15.2% : 3.7% (4.1) | 15.8% : 3.3% |
| Item fit (infit MnSq)γ | <1.5 | **CN45 (1.89), CN4 (1.70), CN46 (1.55), CN3 (1.53), CN15 (1.51)** | None |
| Item fit (outfit MnSq)γ | <1.5 | **CN45 (2.43), CN4 (1.81), CN3 (1.70)** | **CN2 (1.53), CN6 (1.52)** |
| DIF‡ | <1.0 logits and p<0.05 | **Age: CN3 (-1.30), CN4 (-1.22)**  **Ethnicity: CN15 (-1.68 & -1.06)** | None |
| Measurement range | >2.0 logits | 3.28 | 3.59 |
| Missing data^ | <25% | **CN16 (91.2%), CN20 (26.3%), CN38, CN42 (25.6%)** | **CN20 (26.3%), CN38, CN42 (25.6%)** |
| Targeting, difference between person & item means | <1.0 logits | **2.16** | **2.22** |
| TIF |  | 42.5 | 34.4 |
| CN3=Losing your balance; CN4=Falling; CN15=Your ability to follow your religious practices; CN16=Not being able to complete your education; CN20=Not being able to see your children or grandchildren grow up; CN38=Diabetes affecting your personal relationships; CN42=Your diabetes limiting your children's prospects in life; CN45=Having to tell people that you have diabetes; CN46=How people react to your diabetes  *Ceiling effect: 6 (2.0%)  φCategory 2 ‘Moderately’ underused compared to other categories  †’Safety’ items loading >0.4; Disattenuated correlations: Dimensions 1-2 = 0.85; 2-3 = 1.0  γHigh item misfit for 4 items was resolved by diagnosing misfit through unpredictable individual responses (total n=5) and giving errant responders a weighting of 0 – see methods  §Deleted 3 items for misfit and DIF (CN3, CN4 and CN15); 2 items for misfit and high loading contributing to high eigenvalue (CN45 and CN46); and 1 item for >90% missing data (CN16).  ^Retained the remaining items with high missing data as content important and close to the cut-off  †’Safety’ items loading >0.4; Disattenuated correlations: Dimensions 1-2 = 0.85; 2-3 = 1.0  ‡DIF was assessed for age group (≤55 vs. >55 years), gender, language of interview (English vs. Mandarin/Malay), DM duration (≤13 vs. >13 years), No. DM complications (0-1 vs. >1), ethnicity (Chinese, Malay, Indian)  LID >0.2 = 43 pairs of 861, 5.0%  Bolded values represent poor fit to the Rasch model  DIF=Differential item functioning; LID=Local item dependency; PCA=Principal components analysis; TIF=Test information function | | | |

| Additional File 8: Psychometric properties of the DM management domain (N=297) | | | | | | |
| --- | --- | --- | --- | --- | --- | --- |
| Parameters | Rasch model | **First iteration (all items) (n=297)** | “DM Management-Convenience” (n=297) | Final iteration (n=283)*^1;^ §^1^ | “DM Management-Concerns” (n=297) | Final iteration (n=289)*^2;^ §^2^ |
| Disordered thresholds | No | Noφ | No | No | No | No |
| No. of items | - | 63 | 30 | 28 | 33 | 28 |
| Person separation index | >2.0 | 3.10 | 2.09 | 2.45 | 2.61 | 3.00 |
| Person reliability | >0.8 | 0.91 | 0.81 | 0.86 | 0.87 | 0.90 |
| Item separation index | >3.0 | 5.88 | 5.49 | 5.68 | 5.81 | 5.74 |
| PCA, variance by 1^st^ factor | >50% | **44.1%**†^1^ | **47.3%** | **48.2%**†^2^ | 50.1% | 51.4% |
| PCA, Eigenvalue for 1^st^ contrast | <3.0 | **5.8**†^1^ | **3.3** | **3.3**†^2^ | 2.9 | 2.9 |
| PCA, ratio % raw variance explained by items: % raw variance explained by 1^st^ contrast | Larger ratio = better | 15.2% : 5.2% (2.9) | 13.4% : 5.8% (2.3) | 12.9% : 6.2% (2.1) | 15.3% : 4.3% (3.3) | 13.9% : 5.0% (2.8) |
| Item fit (infit MnSq) | <1.5 | **DM19 (1.85), DM41 (1.59), DM43 (1.62), DM36 (1.55)** | **DM19 (2.01), DM30 (1.51)** | Noneγ^1^ | **DM36 (1.55), DM58 (1.55), DM43 (1.70), DM41 (1.69)** | **DM36 (1.54), DM58 (1.53)**γ^2^ |
| Item fit (outfit MnSq) | <1.5 | **DM19 (1.72); DM41 (1.65), DM40 (1.62), DM36 (1.59), DM25 (1.53), DM35 (1.51)** | **DM19 (2.08)**  **DM16 (1.70)** | Noneγ^1^ | **DM35 (1.96), DM36 (1.82), DM41 (1.66), DM40 (1.69), DM58 (1.82), DM54 (1.73)** | **DM35 (1.57)**γ^2^ |
| DIF‡ | <1.0 logits and p<0.05 | None | **Gender: DM19 (-1.20)** | None | **Gender: DM40 (1.52)**  **Ethnicity: DM54 (1v2, 1.13)**  **Ethnicity: DM55 (1v2, 1.01)** | None |
| Measurement range | >2.0 logits | 2.60 | 2.31 | 2.45 | 1.93 | 2.00 |
| Missing data^ | <25% | **DM25 (60.0%), DM26 (72.1%), DM27-29 (70.0%), DM30 (70.3%), DM42-43 (69.0%), DM44 (68.0%), DM45 (66.0%), DM52 (32.0%), DM53 (36.7%)** | As per first iteration | As per first iteration | As per first iteration | As per first iteration |
| Targeting, difference between person & item means | <1.0 logits | **1.52** | **2.17** | **2.03** | **1.39** | **1.30** |
| TIF | >10 | 69.6 | 29.6 | 26.3 | 35.7 | 28.1 |
| DM16=Getting the right amount and type of exercise for your diabetes; DM19=Having to swallow large pills for diabetes; DM25=Administering your insulin; DM30= Not being able to wear the clothes that you want due to having to inject insulin; DM35=Getting the right medication and dosage for your diabetes; DM36=Running out of diabetes medication; DM40=Your sex life being affected as a result of your diabetes medications; DM41=Having to take insulin injections in the future; DM43=Not measuring your insulin dose accurately; DM54=People nagging you about your diabetes management plans; DM55=Being criticised about the way you manage your diabetes; DM58=Having to prick your finger to self-monitor your blood sugar level  *^1^Ceiling effect: 15 (5.1%); *^2^Ceiling effect: 8 (2.7%)  φThe hardest to endorse (i.e. ‘worst’) two categories both underused compared to other categories.  γ^1^High item misfit for 3 items was resolved by diagnosing misfit through unpredictable individual responses (total n=8) and giving errant responders a weighting of 0 – see methods; γ^2^High item misfit for 3 items was resolved by diagnosing misfit through unpredictable individual responses (total n=19)  †^1^’Convenience’ items loading >0.4, Disattenuated correlations: Dimensions 1-2 = 0.95; 2-3 = 0.87; ^2^Disattenuated correlations: Dimensions 1-2/2-3 = 1.0  §^1^Deleted 1 item (DM19) for misfit and DIF, and 1 item for misfit (DM16); §^2^ Deleted 3 items for misfit and DIF (DM40, DM54, DM55) and 2 items for misfit (DM41, DM43)  ^These items cannot be answered if the patient does not use insulin. During CAT administration, these items will be not be administered if the patient responds ‘No’ to an initial screening question “Do you use insulin to manage your diabetes?”, and therefore they will not contribute to response burden if not applicable.  ‡DIF was assessed for age group (≤55 vs. >55 years), gender, language of interview (English vs. Mandarin/Malay), DM duration (≤13 vs. >13 years), No. DM complications (0-1 vs. >1), ethnicity (Chinese, Malay, Indian)  DM-CV: LID >0.2 = 24 pairs of 378, 6.3%); DM-CN: LID>0.2 = 18 pairs of 378, 4.8%  Bolded values represent poor fit to the Rasch model.  DIF=Differential item functioning; DM=Diabetes Mellitus; LID=Local item dependency; PCA=Principal components analysis; RS=Rating scale; TIF=Test information function | | | | | | |

| Additional File 9: Psychometric properties of the Work domain (N=178) | | | |
| --- | --- | --- | --- |
| Parameters | Rasch model | First iteration (n=178) | **Final iteration*§**  (n=144) |
| Disordered thresholds | No | No | No |
| No. of items | - | 24 | 23 |
| Person separation index | >2.0 | **1.80** | 2.63 |
| Person reliability | >0.8 | **0.76** | 0.87 |
| Item separation index | >3.0 | 4.81 | 5.13 |
| PCA, variance by 1^st^ factor | >50% | 59.4% | 61.6% |
| PCA, Eigenvalue for 1^st^ contrast | <3.0 | **3.6**† | **3.6**† |
| PCA, ratio % raw variance explained by items: % raw variance explained by 1^st^ contrast | Larger ratio = better | 12.5% : 6.1% (2.0) | 9.4% : 6.1% (1.5) |
| Item fit (infit MnSq) | <1.5 | **WK19 (2.09). WK23 (1.51)** | Noneγ |
| Item fit (outfit MnSq) | <1.5 | **WK19 (2.83), WK18 (1.81)** | Noneγ |
| DIF‡ | <1.0 logits and p<0.05 | **DM Complications: WK17 (1.03)**  **Ethnicity WK19 (-1.58 Malay vs. Indian)** | None |
| Measurement range | >2.0 logits | 2.98 | 3.32 |
| Missing data | <25% | None | None |
| Targeting, difference between person & item means | <1.0 logits | **2.91** | **2.44** |
| TIF | >10.0 | 23.0 | 21.4 |
| WK17=Losing your job; WK18=Needing to take sick leave or time off work due to DM; WK19=Not being able to find diabetes-friendly food at work; WK23=Planning your work schedule around your diabetes management regime  *Of the 297 patients, 178 (60%) answered the Work domain. Ceiling effect: 34 (19.1%)  γItem misfit for 2 items was resolved by diagnosing misfit through unpredictable individual responses (total n=5) and giving errant responders a weighting of 0 – see methods  †Disattenuated correlations = 1.0  §Deleted 1 item for misfit and DIF (WK19)  ‡DIF was assessed for age group (≤55 vs. >55 years), gender, language of interview (English vs. Mandarin/Malay), DM duration (≤13 vs. >13 years), No. DM complications (0-1 vs. >1), ethnicity (Chinese, Malay, Indian)  LID >0.2 = 40 pairs of 253, 15.8%  Bolded values represent poor fit to the Rasch model.  DIF=Differential item functioning; LID=Local item dependency; PCA=Principal components analysis; RS=Rating scale; TIF=Test information function | | | |

| **Additional File 10:** Category Probability Curves, Test Information Function, and Person-Item Map of the Activity limitation (AL) domain | | |
| --- | --- | --- |
|  |    | MEASURE PERSON - MAP - ITEM  <more>\|<rare>  6 ############ +  \|  \|  \|  \|  ########### S\|  5 +  .## \|  \|  ####### \|  \|  ## \|  4 .#### +  \|  .######### M\|  .###### \|  # \|  .####### \|  3 .## +  .## \|  .### \|  ###### \|  ### \| D-AL12  .# S\|  2 .## +T  .### \|  .## \|  .# \|  . \| D-AL18 D-AL8  .# \| D-AL23  1 . +S  # \| D-AL3  # \|  T\| D-AL7  . \| D-AL22  . \| D-AL1 D-AL11  0 +M D-AL6  . \| D-AL15 D-AL19 D-AL21  . \| D-AL20  \|  \|  \| D-AL13 D-AL2 D-AL9  -1 +S D-AL10 D-AL14 D-AL16  \|  \|  \|  \|  \| D-AL5  -2 +T  <less>\|<freq> |
| **Category probability curves of the AL domain.** Thresholds were ordered both before and after amendments. **Response Cateogory**: Red Curve: “Unable to do because of my diabetes”; Blue Curve: “A lot”; Pink Curve: “Quite a bit”; Black Curve: “A little”; Green curve: “None”. | **Test information curve of the AL domain.** A higher level of information indicates greater measurement precision at that point along the scale. Before scale amendments (top), the value was 17.0. After scale amendments (bottom), the TIF value was 15.6. | **Person–item map for the AL domain after amendments.** To the left of the dashed line are the participants, represented by ‘#’ and ‘.’ signifying 3 and 1-2 participants, respectively, and on the right are the items, denoted by their item number. Participants with ‘better’ physical functioning and the most ‘difficult’ items are near the top, and participants ‘worse’ physical functioning and the least ‘difficult’ items are near the bottom. This figure shows that items in the AL domain are not well targeted to participant ability level with >1 logit difference between the mean of person AL and mean of item difficulty. There are several items near the bottom of the figure that are too easy for this particular patient sample. |

| **Additional File 11:** Category Probability Curves, Test Information Function, and Person-Item Map of the Symptoms (SY) domain | | |
| --- | --- | --- |
|    |    | MEASURE PERSON - MAP - ITEM  <more>\|<rare>  5 +  .###### T\|  \|  \|  \|  \|  ############ \|  4 +  \|  ######### S\|  \|  .######## \|  .###### \|  \|  3 .###### +  ####### \|  ####### M\|  .######### \|  .##### \|  .########### \|  ######### \|  2 .##### +  ##### \|  ### \|  #### S\|  ##### \|  .#### \|  .## \|T  1 # + D-SY12 D-SY17 D-SY18  # \|  . \| D-SY11 D-SY2 D-SY21  . \|S D-SY13  . T\| D-SY28 D-SY3 D-SY6  # \| D-SY15 D-SY23 D-SY9  . \|  0 . +M D-SY10 D-SY27  # \| D-SY1 D-SY20 D-SY32 D-SY7  # \| D-SY19 D-SY5 D-SY8  \| D-SY25 D-SY26 D-SY30  \|S D-SY14 D-SY29  \| D-SY33  \| D-SY22 D-SY4  -1 +  \|T  \| D-SY24  \|  \|  \|  \|  -2 +  <less>\|<freq>  EACH "#" IS 2: EACH "." IS 1 |
| **Category probability curves of the SY domain.** Thresholds were ordered both before and after amendments. **Response Cateogory**: Red Curve: “Extremely”; Blue Curve: “Very”; Pink Curve: “Moderately”; Black Curve: “A little bit”; Green curve: “Not at all”. | **Test information curve of the SY domain.** A higher level of information indicates greater measurement precision at that point along the scale. Before scale amendments (top), the TIF value was 32.1. After scale amendments (bottom), the TIF value was 28.9. | **Person–item map for the SY domain after amendments.** To the left of the dashed line are the participants, represented by ‘#’ and ‘.’ signifying 2 and 1 participants, respectively, and on the right are the items, denoted by their item number. Participants with ‘less’ symptomology and the most ‘difficult’ items are near the top, and participants with ‘more’ symptomology and the least ‘difficult’ items are near the bottom. This figure shows that items in the SY domain are not well targeted to participant ability level with >1 logit difference between the mean of person SY and mean of item difficulty. There are several items near the bottom of the figure that are too easy for this particular patient sample. |

| **Additional File 12:** Category Probability Curves, Test Information Function, and Person-Item Map of the Diet (DT) domain | | |
| --- | --- | --- |
| A)   B)   C)   D)  |    | MEASURE PERSON - MAP - ITEM  <more>\|<rare>  5 +  \|  \|  \|  \|  \|  .#### \|  4 ## T+  \|  .# \|  ## \|  .## \|  \|  \|  3 .#### +  .## S\|  .##### \|  .####### \|  .#### \|  ## \|  .######### \|  2 .## +  .####### \|  ######## \|  ###### M\|  .######## \|  .##### \|  .##### \|  1 ####### +  .### \|T  ###### \|  ###### \| DT17  ## S\|S DT1 DT4 DT9  ### \| DT18 DT2  #### \| DT10 DT12 DT3 DT6 DT8  0 .### +M DT14 DT5  .## \|  # \| DT7  # \|S DT16  # \| DT11  . T\|  . \|T DT15  -1 + DT13  . \|  \|  \|  . \|  \|  \|  -2 +  . \|  \|  \|  \|  \|  \|  -3 +  <less>\|<freq>  EACH "#" IS 2: EACH "." IS 1 |
| **Category probability curves of the DT domain.** Thresholds are ordered but uneven both before (A & B) and after (C & D) amendment, with the blue response category considerably more likely than the others to be utilised in patients around the -1.5 logit ability range. **Response Cateogory A & B**: Red Curve: “Too much trouble to do”; Blue Curve: “A lot”; Pink Curve: “A moderate amount”; Black Curve: “A little bit”; Green curve: “Not at all”.  **Response Cateogory C & D**: Red Curve: “Extremely”; Blue Curve: “Very”; Pink Curve: “Moderately”; Black Curve: “A little bit”; Green curve: “Not at all”. | **Test information curve of the DT domain.** A higher level of information indicates greater measurement precision at that point along the scale. Before scale amendments (top), the TIF value was 18.1. After scale amendments (bottom), the TIF value was 17.5. | **Person–item map for the DT domain after amendments.** To the left of the dashed line are the participants, represented by ‘#’ and ‘.’ signifying 2 and 1participants, respectively, and on the right are the items, denoted by their item number. Participants with less dietary issues and the most ‘difficult’ items are near the top, and participants more dietary issues and the least ‘difficult’ items are near the bottom. This figure shows that items in the DT domain are not well targeted to participant ability level with >1 logit difference between the mean of person DT and mean of item difficulty. There are several items near the bottom of the figure that are too easy for this particular patient sample. |

| **Additional File 13:** Category Probability Curves, Test Information Function, and Person-Item Map of the Emotional (EM) domain | | |
| --- | --- | --- |
| A)    B)    C)    D)   |    | MEASURE PERSON - MAP - ITEM  <more>\|<rare>  6 +  ######### \|  \|  \|  \|  ######## S\|  5 +  \|  .##### \|  .## \|  \|  .### \|  4 #### +  #### \|  ### M\|  #### \|  ## \|  .## \|  3 .## +  .# \|  .## \|  ### \|  .### \|  . S\|  2 ## +  ## \|  .## \|  . \|T D-EM5  . \|  . \|  1 . + D-EM40 D-EM45 D-EM46  # \| D-EM1 D-EM8  . T\|S D-EM10 D-EM16 D-EM22 D-EM7  . \| D-EM15 D-EM20 D-EM21 D-EM26 D-EM4  . \| D-EM13 D-EM29 D-EM3 D-EM42 D-EM47  \| D-EM27 D-EM44  0 +M D-EM11 D-EM2 D-EM28  \| D-EM17 D-EM32  \| D-EM19 D-EM36  \| D-EM14 D-EM37  \|S D-EM23 D-EM24 D-EM25 D-EM30 D-EM39  \| D-EM33  -1 +  \| D-EM38  \| D-EM12 D-EM34 D-EM35  \|T D-EM6  \|  \|  -2 +  \|  \|  \|  . \|  \|  -3 +  <less>\|<freq>  EACH "#" IS 3: EACH "." IS 1 TO 2 |
| **Category probability curves of the EM domain.** Thresholds are ordered but uneven for the first rating scale, both before (A) and after (C) amendment, with the blue response category considerably more likely than the others to be utilised in patients around the -1.5 logit ability range. **Response Cateogories**: Red Curve: “All of the time”; Blue Curve: “Most of the time”; Pink Curve: “Some of the time”; Black Curve: “A little of the time”; Green curve: “None of the time”. | **Test information curve of the EM domain.** A higher level of information indicates greater measurement precision at that point along the scale. Before scale amendments (top), the TIF value was 36.4. After scale amendments (bottom), the TIF value was 29.2 | **Person–item map for the EM domain after scale amendments.** To the left of the dashed line are the participants, represented by ‘#’ and ‘.’ signifying 3 and 1-2 participants, respectively, and on the right are the items, denoted by their item number. Participants with ‘better’ emotional well-being and the most ‘difficult’ items are near the top, and participants ‘worse’ emotional well-being and the least ‘difficult’ items are near the bottom. This figure shows that items in the EM domain are not well targeted to participant ability level with >1 logit difference between the mean of person EM and mean of item difficulty. There are several items near the bottom of the figure that are too easy for this particular patient sample. |

| **Additional File 14:** Category Probability Curves, Test Information Function, and Person-Item Map of the Concerns (CN) domain | | |
| --- | --- | --- |
| A)    B)    C)    D)   |    | MEASURE PERSON - MAP - ITEM  <more>\|<rare>  6 +  \|  \|  # \|  . \|  \|  5 +  T\|  ### \|  \|  .### \|  . \|  4 .#### +  .### \|  #### \|  ### S\|  .###### \|  ######## \|  3 .#### +  .######## \|  .##### \|  ########## \|  ######### \|  #### M\|  2 .####### +  ######## \|T D-CN7  .##### \|  .##### \|  ##### \| D-CN30 D-CN31  #### \| D-CN41  1 ###### S+ D-CN6  ###### \|S D-CN19 D-CN33 D-CN34 D-CN40  ## \| D-CN10 D-CN11 D-CN12 D-CN8  ## \| D-CN13 D-CN2 D-CN9  .# \| D-CN1 D-CN42  # \| D-CN17 D-CN20 D-CN21 D-CN36 D-CN37  0 .# +M D-CN18 D-CN32 D-CN35  .### \| D-CN29  ## T\| D-CN14 D-CN39  # \|  \| D-CN22 D-CN24 D-CN28  . \|S  -1 + D-CN23 D-CN43 D-CN47 D-CN48  . \| D-CN38 D-CN5  \| D-CN44  \| D-CN25  \|  \|T D-CN26 D-CN27  -2 +  \|  . \|  \|  \|  \|  -3 +  <less>\|<freq>  EACH "#" IS 2: EACH "." IS 1 |
| **Category probability curves of the CN domain.** Thresholds are ordered but uneven for both rating scales, both before (A & B) and after (C & D) amendment, with the blue response category considerably more likely than the others to be utilised in patients around the -1.5 logit ability range. **Response Cateogory**: Red Curve: “Extremely”; Blue Curve: “Very”; Pink Curve: “Moderately”; Black Curve: “A little bit”; Green curve: “Not at all”. | **Test information curve of the CN domain.** A higher level of information indicates greater measurement precision at that point along the scale. Before scale amendments (top), the TIF value was 42.5. After scale amendments (bottom), the TIF value was 34.4. | **Person–item map for the CN domain after scale amendments.** To the left of the dashed line are the participants, represented by ‘#’ and ‘.’ signifying 2 and 1 participants, respectively, and on the right are the items, denoted by their item number. Participants with fewer concerns and the most ‘difficult’ items are near the top, and participants more concerns and the least ‘difficult’ items are near the bottom. This figure shows that items in the CN domain are not well targeted to participant ability level with >1 logit difference between the mean of person CN and mean of item difficulty. There are several items near the bottom of the figure that are too easy for this particular patient sample. |

| **Additional File 15:** Category Probability Curves, Test Information Function, and Person-Item Map of the DMn–Concerns (DMn-CN) domain | | |
| --- | --- | --- |
|    |    | MEASURE PERSON - MAP - ITEM  <more>\|<rare>  5 +  \|  \|  \|  \|  . \|  . \|  \|  4 +  \|  .# \|  \|  . \|  # T\|  # \|  . \|  3 ## +  ## \|  # \|  .# \|  ##### \|  ### S\|  ### \|  ###### \|  2 .###### +  .#### \|  .###### \|  .##### \|  ###### \|  .########### \|  .####### M\|  .######## \|T D-DM59  1 #### +  ###### \| D-DM48  .##### \| D-DM53 D-DM60 D-DM61  .## \|  .##### \|S D-DM52 D-DM62  ### \| D-DM31 D-DM45  ### S\| D-DM39  .#### \| D-DM33 D-DM38 D-DM46 D-DM47  0 .##### +M  .# \| D-DM35 D-DM37 D-DM49 D-DM63  #### \| D-DM50  ## \| D-DM51 D-DM57  . \|S  . \| D-DM32 D-DM36 D-DM56  T\| D-DM34 D-DM42  . \| D-DM44 D-DM58  -1 . +  \|T  \|  . \|  \|  \|  \|  . \|  -2 +  \|  . \|  \|  \|  \|  \|  \|  -3 +  <less>\|<freq>  EACH "#" IS 2: EACH "." IS 1 |
| **Category probability curves of the DMn-CN domain.** Thresholds were ordered but bunched before amendments. Thresholds were somewhat improved after scale amendments. **Response Cateogory**: Red Curve: “Extremely”; Blue Curve: “Very”; Pink Curve: “Moderately”; Black Curve: “A little bit”; Green curve: “Not at all”. | **Test information curve of the DMn-CN domain.** A higher level of information indicates greater measurement precision at that point along the scale. Before scale amendments (top), the TIF value was 35.7. After scale amendments (bottom), the TIF value was 28.1. | **Person–item map for the DMn-CN domain after amendments.** To the left of the dashed line are the participants, represented by ‘#’ and ‘.’ signifying 2 and 1 participants, respectively, and on the right are the items, denoted by their item number. Participants with less DM management concerns and the most ‘difficult’ items are near the top, and participants more DM management concerns and the least ‘difficult’ items are near the bottom. This figure shows that items in the DMn-CN domain are not well targeted to participant ability level with >1 logit difference between the mean of person DMn-CN and mean of item difficulty. There are several items near the bottom of the figure that are too easy for this particular patient sample. |

| **Additional File 16:** Category Probability Curves, Test Information Function, and Person-Item Map of the DMn–Convenience (DMn-CV) domain | | |
| --- | --- | --- |
|    |    | MEASURE PERSON - MAP - ITEM  <more>\|<rare>  5 +  \|  ## \|  .##### \|  # T\|  . \|  \|  4 .# +  ##### \|  \|  # \|  .#### \|  ## S\|  ##### \|  3 .### +  .### \|  .# \|  .#### \|  ##### \|  ######### \|  .##### \|  2 ###### M+  .############ \|  .####### \|  ###### \|  ######## \|T  .## \|  ###### \|  1 ####### + D-DM1 D-DM10 D-DM3 D-DM6  .###### S\| D-DM11  .#### \|S D-DM12 D-DM13 D-DM14 D-DM5  ## \| D-DM26  .# \|  ## \| D-DM2 D-DM8  ## \| D-DM9  0 # +M D-DM25  . \| D-DM15 D-DM18 D-DM20  .# T\| D-DM4  \| D-DM24 D-DM7  \|  \|S D-DM17 D-DM21 D-DM22 D-DM23 D-DM29  # \|  -1 +  \| D-DM27  \| D-DM30  \|T D-DM28  \|  \|  \|  -2 +  \|  \|  . \|  \|  \|  \|  -3 +  <less>\|<freq>  EACH "#" IS 2: EACH "." IS 1 |
| **Category probability curves of the DMn-CV domain.** Thresholds were ordered both before and after amendments. **Response Cateogory**: Red Curve: “Extremely”; Blue Curve: “Very”; Pink Curve: “Moderately”; Black Curve: “A little bit”; Green curve: “Not at all”. | **Test information curve of the DMn-CV domain.** A higher level of information indicates greater measurement precision at that point along the scale. Before scale amendments (top), the TIF value was 29.6. After scale amendments (bottom), the TIF value was 26.3. | **Person–item map for the DMn-CV domain after amendments.** To the left of the dashed line are the participants, represented by ‘#’ and ‘.’ signifying 2 and 1 participants, respectively, and on the right are the items, denoted by their item number. Participants with less DM management inconvenience and the most ‘difficult’ items are near the top, and participants more DMn-CV management inconvenience and the least ‘difficult’ items are near the bottom. This figure shows that items in the DMn-CV domain are not well targeted to participant ability level with >1 logit difference between the mean of person DMn-CV and mean of item difficulty. There are several items near the bottom of the figure that are too easy for this particular patient sample. |

| **Additional File 17:** Category Probability Curves, Test Information Function, and Person-Item Map of the Work (WK) domain | | |
| --- | --- | --- |
| A)    B)    C)    D)   |    | MEASURE PERSON - MAP - ITEM  <more>\|<rare>  5 +  \|  ##### \|  \|  \|  \|  4 ######## S+  \|  \|  .##### \|  ## \|  # \|  3 ####### +  ## \|  # \|  ### M\|  .## \|  .##### \|  2 .### +  .## \|  ## \|T  .## \| D-WK14  # \|  .# \| D-WK13 D-WK17  1 # S+  .# \|S D-WK18  # \| D-WK15 D-WK16 D-WK5  . \| D-WK1  # \|  # \| D-WK11 D-WK12 D-WK3 D-WK6  0 . +M D-WK2  . \| D-WK4 D-WK7  . \|  .# T\| D-WK10 D-WK22 D-WK8  \|  \|S D-WK23 D-WK9  -1 +  \| D-WK20  \| D-WK24  \|  \|T  \| D-WK21  -2 +  \|  \|  \|  \|  \|  -3 +  \|  \|  \|  \|  \|  -4 # +  <less>\|<freq>  EACH "#" IS 2: EACH "." IS 1 |
| **Category probability curves of the WK domain.** Thresholds are ordered but uneven for the second rating scales, both before (B) and after (D) amendment, with the blue response category considerably more likely than the others to be utilised in patients around the -2.0 logit ability range. **Response Cateogory RS1**: Red Curve: “Extremely”; Blue Curve: “Very”; Pink Curve: “Moderately”; Black Curve: “A little bit”; Green curve: “Not at all”. **Response Cateogory RS2:** Red Curve: “Unable to do because of my diabetes”; Blue Curve: “A lot”; Pink Curve: “Quite a bit”; Black Curve: “A little”; Green curve: “None”. | **Test information curve of the WK domain.** A higher level of information indicates greater measurement precision at that point along the scale. Before scale amendments (top), the TIF value was 23.0. After scale amendments (bottom), the TIF value was 21.4. | **Person–item map for the WK domain after scale amendments.** To the left of the dashed line are the participants, represented by ‘#’ and ‘.’ signifying 2 and 1 participants, respectively, and on the right are the items, denoted by their item number. Participants with fewer work issues and the most ‘difficult’ items are near the top, and participants more work issues and the least ‘difficult’ items are near the bottom. This figure shows that items in the WK domain are not well targeted to participant ability level with >1 logit difference between the mean of person WK and mean of item difficulty. There are several items near the bottom of the figure that are too easy for this particular patient sample. |

| **Additional File 18:** CAT simulation results for the Symptoms domain at two precision levels | | | | | | | | | | |
| --- | --- | --- | --- | --- | --- | --- | --- | --- | --- | --- |
| *SEM 0.3 (high precision)* | | | | | | | | | | |
| **Measure** | **D1** | **D2** | **D3** | **D4** | **D5** | **D6** | **D7** | **D8** | **D9** | **D10** |
| Mean Theta | -1.833 | -1.036 | -0.607 | -0.337 | -0.083 | 0.184 | 0.459 | 0.759 | 1.11 | 1.754 |
| RMSE | 0.729 | 0.683 | 0.886 | 1.119 | 0.679 | 0.514 | 0.659 | 0.862 | 0.869 | 1.055 |
| Mean bias | -0.15 | -0.034 | -0.091 | -0.378 | -0.093 | -0.21 | -0.288 | -0.253 | -0.081 | 0.066 |
| Mean test length | 21.38 | 14.99 | 14.1 | 14.17 | 12.68 | 12.08 | 12.09 | 13.16 | 15.48 | 20.85 |
| Mean standard error | 0.34 | 0.298 | 0.303 | 0.311 | 0.295 | 0.292 | 0.293 | 0.293 | 0.294 | 0.339 |
| Proportion stop rule satisfied | 0.75 | 0.92 | 0.93 | 0.92 | 0.98 | 1 | 1 | 1 | 1 | 0.74 |
| Number of simulees | 100 | 100 | 100 | 100 | 100 | 100 | 100 | 100 | 100 | 100 |
| *SEM 0.387 (moderate precision)* | | | | | | | | | | |
| Mean Theta | -1.833 | -1.036 | -0.607 | -0.337 | -0.083 | 0.184 | 0.459 | 0.759 | 1.11 | 1.754 |
| RMSE | 0.89 | 0.749 | 0.881 | 1.129 | 0.693 | 0.545 | 0.661 | 0.849 | 0.896 | 1.074 |
| Mean bias | -0.103 | -0.017 | -0.073 | -0.362 | -0.078 | -0.223 | -0.303 | -0.332 | -0.072 | 0.053 |
| Mean test length (items) | 15.41 | 9.46 | 8.7 | 9.09 | 7.52 | 7.14 | 7.24 | 7.68 | 9.21 | 14.11 |
| Mean standard error | 0.405 | 0.379 | 0.387 | 0.393 | 0.381 | 0.38 | 0.379 | 0.376 | 0.377 | 0.403 |
| Proportion stop rule satisfied | 0.82 | 0.98 | 0.97 | 0.93 | 0.99 | 1 | 1 | 1 | 1 | 0.83 |
| Number of simulees | 100 | 100 | 100 | 100 | 100 | 100 | 100 | 100 | 100 | 100 |
| CAT=Computerized adaptive test; D=decile; RMSE=Root mean square error; SEM=Standard error of measurement | | | | | | | | | | |

| **Additional File 19:** CAT simulation results for the Diet domain at two precision levels | | | | | | | | | | |
| --- | --- | --- | --- | --- | --- | --- | --- | --- | --- | --- |
| *SEM 0.3 (high precision)* | | | | | | | | | | |
| **Measure** | **D1** | **D2** | **D3** | **D4** | **D5** | **D6** | **D7** | **D8** | **D9** | **D10** |
| Mean Theta | -1.833 | -1.036 | -0.607 | -0.337 | -0.083 | 0.184 | 0.459 | 0.759 | 1.11 | 1.754 |
| RMSE | 0.916 | 1.125 | 1.124 | 1.243 | 0.869 | 0.743 | 0.777 | 0.857 | 0.912 | 1.295 |
| Mean bias | -0.349 | -0.24 | -0.232 | -0.051 | -0.08 | 0.111 | 0.064 | -0.015 | 0.01 | 0.265 |
| Mean test length | 18.41 | 15.72 | 14.19 | 13.28 | 13.19 | 12.56 | 12.58 | 13.41 | 14.62 | 15.94 |
| Mean standard error | 0.428 | 0.359 | 0.34 | 0.333 | 0.304 | 0.296 | 0.296 | 0.302 | 0.316 | 0.471 |
| Proportion stop rule satisfied | 0.19 | 0.68 | 0.8 | 0.87 | 0.9 | 0.98 | 0.96 | 0.84 | 0.73 | 0.54 |
| Number of simulees | 100 | 100 | 100 | 100 | 100 | 100 | 100 | 100 | 100 | 100 |
| *SEM 0.387 (moderate precision)* | | | | | | | | | | |
| Mean Theta | -1.833 | -1.036 | -0.607 | -0.337 | -0.083 | 0.184 | 0.459 | 0.759 | 1.11 | 1.754 |
| RMSE | 0.908 | 1.127 | 1.129 | 1.269 | 0.822 | 0.754 | 0.787 | 0.863 | 0.873 | 1.29 |
| Mean bias | -0.332 | -0.181 | -0.103 | -0.023 | -0.085 | 0.132 | 0.168 | 0.015 | 0.006 | 0.305 |
| Mean test length (items) | 15.2 | 10.67 | 9.17 | 8.6 | 8.1 | 7.64 | 7.79 | 8.44 | 9.75 | 12.53 |
| Mean standard error | 0.457 | 0.418 | 0.409 | 0.409 | 0.383 | 0.377 | 0.375 | 0.378 | 0.381 | 0.519 |
| Proportion stop rule satisfied | 0.57 | 0.81 | 0.86 | 0.9 | 0.94 | 0.99 | 0.99 | 0.97 | 0.91 | 0.63 |
| Number of simulees | 100 | 100 | 100 | 100 | 100 | 100 | 100 | 100 | 100 | 100 |
| CAT=Computerized adaptive test; D=decile; RMSE=Root mean square error; SEM=Standard error of measurement | | | | | | | | | | |

| **Additional File 20:** CAT simulation results for the Emotional domain at two precision levels | | | | | | | | | | |
| --- | --- | --- | --- | --- | --- | --- | --- | --- | --- | --- |
| *SEM 0.3 (high precision)* | | | | | | | | | | |
| **Measure** | **D1** | **D2** | **D3** | **D4** | **D5** | **D6** | **D7** | **D8** | **D9** | **D10** |
| Mean Theta | -1.833 | -1.036 | -0.607 | -0.337 | -0.083 | 0.184 | 0.459 | 0.759 | 1.11 | 1.754 |
| RMSE | 0.888 | 0.779 | 0.959 | 1.315 | 0.712 | 0.829 | 0.97 | 0.96 | 0.734 | 0.967 |
| Mean bias | -0.082 | -0.037 | 0.003 | -0.062 | 0.031 | 0.232 | 0.247 | 0.188 | 0.189 | -0.005 |
| Mean test length | 24.72 | 19.76 | 18.26 | 18.83 | 16.65 | 16.09 | 16.43 | 16.24 | 15.96 | 20.17 |
| Mean standard error | 0.302 | 0.298 | 0.297 | 0.298 | 0.294 | 0.295 | 0.296 | 0.296 | 0.296 | 0.3 |
| Proportion stop rule satisfied | 0.9 | 0.95 | 0.97 | 0.95 | 1 | 1 | 1 | 1 | 1 | 0.95 |
| Number of simulees | 100 | 100 | 100 | 100 | 100 | 100 | 100 | 100 | 100 | 100 |
| *SEM 0.387 (moderate precision)* | | | | | | | | | | |
| Mean Theta | -1.833 | -1.036 | -0.607 | -0.337 | -0.083 | 0.184 | 0.459 | 0.759 | 1.11 | 1.754 |
| RMSE | 0.908 | 0.781 | 0.9 | 1.248 | 0.75 | 0.852 | 1.017 | 1.002 | 0.853 | 0.984 |
| Mean bias | -0.131 | 0.007 | 0.048 | -0.007 | -0.037 | 0.184 | 0.243 | 0.188 | 0.201 | 0.083 |
| Mean test length (items) | 15.04 | 11.58 | 10.85 | 11.1 | 10.26 | 9.93 | 10.41 | 10.09 | 9.77 | 13.53 |
| Mean standard error | 0.38 | 0.377 | 0.376 | 0.377 | 0.374 | 0.374 | 0.375 | 0.378 | 0.38 | 0.378 |
| Proportion stop rule satisfied | 1 | 1 | 1 | 1 | 1 | 1 | 1 | 1 | 1 | 0.96 |
| Number of simulees | 100 | 100 | 100 | 100 | 100 | 100 | 100 | 100 | 100 | 100 |
| CAT=Computerized adaptive test; D=decile; RMSE=Root mean square error; SEM=Standard error of measurement | | | | | | | | | | |

| **Additional File 21:** CAT simulation results for the Concerns domain at two precision levels | | | | | | | | | | |
| --- | --- | --- | --- | --- | --- | --- | --- | --- | --- | --- |
| *SEM 0.3 (high precision)* | | | | | | | | | | |
| **Measure** | **D1** | **D2** | **D3** | **D4** | **D5** | **D6** | **D7** | **D8** | **D9** | **D10** |
| Mean Theta | -1.833 | -1.036 | -0.607 | -0.337 | -0.083 | 0.184 | 0.459 | 0.759 | 1.11 | 1.754 |
| RMSE | 0.789 | 0.736 | 0.728 | 0.995 | 0.598 | 0.781 | 0.694 | 0.705 | 0.795 | 0.937 |
| Mean bias | -0.063 | 0.208 | 0.075 | 0.024 | 0.053 | 0.12 | -0.041 | -0.141 | 0.04 | 0.054 |
| Mean test length | 22.53 | 14.04 | 13.5 | 13.85 | 12.69 | 12.63 | 12.35 | 12.22 | 13.73 | 18.9 |
| Mean standard error | 0.3 | 0.296 | 0.297 | 0.297 | 0.294 | 0.293 | 0.292 | 0.293 | 0.293 | 0.309 |
| Proportion stop rule satisfied | 0.85 | 0.98 | 0.98 | 0.97 | 1 | 1 | 1 | 1 | 1 | 0.87 |
| Number of simulees | 100 | 100 | 100 | 100 | 100 | 100 | 100 | 100 | 100 | 100 |
| *SEM 0.387 (moderate precision)* | | | | | | | | | | |
| Mean Theta | -1.833 | -1.036 | -0.607 | -0.337 | -0.083 | 0.184 | 0.459 | 0.759 | 1.11 | 1.754 |
| RMSE | 0.886 | 0.762 | 0.747 | 0.991 | 0.623 | 0.81 | 0.755 | 0.716 | 0.779 | 0.944 |
| Mean bias | -0.014 | 0.18 | 0.162 | 0.13 | 0.129 | 0.126 | 0.009 | -0.1 | 0.048 | 0.04 |
| Mean test length (items) | 12.77 | 8.77 | 8.53 | 8.35 | 7.77 | 7.84 | 7.71 | 7.56 | 8.12 | 11.36 |
| Mean standard error | 0.375 | 0.372 | 0.372 | 0.375 | 0.376 | 0.375 | 0.376 | 0.375 | 0.377 | 0.384 |
| Proportion stop rule satisfied | 0.97 | 1 | 0.98 | 0.99 | 1 | 1 | 1 | 1 | 1 | 0.95 |
| Number of simulees | 100 | 100 | 100 | 100 | 100 | 100 | 100 | 100 | 100 | 100 |
| CAT=Computerized adaptive test; D=decile; RMSE=Root mean square error; SEM=Standard error of measurement | | | | | | | | | | |

| **Additional File 22:** CAT simulation results for the DM Management-Convenience domain at two precision levels | | | | | | | | | | |
| --- | --- | --- | --- | --- | --- | --- | --- | --- | --- | --- |
| *SEM 0.3 (high precision)* | | | | | | | | | | |
| **Measure** | **D1** | **D2** | **D3** | **D4** | **D5** | **D6** | **D7** | **D8** | **D9** | **D10** |
| Mean Theta | -1.833 | -1.036 | -0.607 | -0.337 | -0.083 | 0.184 | 0.459 | 0.759 | 1.11 | 1.754 |
| RMSE | 0.634 | 0.562 | 0.782 | 1.008 | 0.688 | 0.604 | 0.674 | 0.795 | 0.771 | 1.036 |
| Mean bias | 0.016 | 0.045 | -0.091 | -0.344 | 0.028 | -0.028 | -0.092 | -0.199 | -0.059 | 0.157 |
| Mean test length | 19.15 | 13.31 | 12.79 | 12.96 | 11.71 | 11.5 | 11.55 | 11.98 | 13.85 | 20.3 |
| Mean standard error | 0.322 | 0.295 | 0.302 | 0.307 | 0.295 | 0.293 | 0.294 | 0.294 | 0.295 | 0.366 |
| Proportion stop rule satisfied | 0.73 | 0.95 | 0.96 | 0.94 | 0.99 | 1 | 1 | 1 | 1 | 0.56 |
| Number of simulees | 100 | 100 | 100 | 100 | 100 | 100 | 100 | 100 | 100 | 100 |
| *SEM 0.387 (moderate precision)* | | | | | | | | | | |
| Mean Theta | -1.833 | -1.036 | -0.607 | -0.337 | -0.083 | 0.184 | 0.459 | 0.759 | 1.11 | 1.754 |
| RMSE | 0.746 | 0.682 | 0.739 | 0.985 | 0.707 | 0.717 | 0.726 | 0.873 | 0.754 | 1.038 |
| Mean bias | -0.049 | -0.006 | -0.132 | -0.376 | 0.034 | -0.003 | -0.053 | -0.17 | -0.092 | 0.118 |
| Mean test length (items) | 12.42 | 8.67 | 7.85 | 8.2 | 7.11 | 7.2 | 7.27 | 7.6 | 7.99 | 13.15 |
| Mean standard error | 0.389 | 0.37 | 0.377 | 0.379 | 0.375 | 0.373 | 0.375 | 0.375 | 0.378 | 0.425 |
| Proportion stop rule satisfied | 0.92 | 1 | 0.97 | 0.96 | 1 | 1 | 1 | 1 | 1 | 0.82 |
| Number of simulees | 100 | 100 | 100 | 100 | 100 | 100 | 100 | 100 | 100 | 100 |
| CAT=Computerized adaptive test; D=decile; DM=Diabetes Mellitus; RMSE=Root mean square error;  SEM=Standard error of measurement | | | | | | | | | | |

| **Additional File 23:** CAT simulation results for the DM Management-Concerns domain at two precision levels | | | | | | | | | | |
| --- | --- | --- | --- | --- | --- | --- | --- | --- | --- | --- |
| *SEM 0.3 (high precision)* | | | | | | | | | | |
| **Measure** | **D1** | **D2** | **D3** | **D4** | **D5** | **D6** | **D7** | **D8** | **D9** | **D10** |
| Mean Theta | -1.833 | -1.036 | -0.607 | -0.337 | -0.083 | 0.184 | 0.459 | 0.759 | 1.11 | 1.754 |
| RMSE | 0.803 | 1.002 | 1.106 | 1.282 | 0.83 | 0.568 | 0.633 | 0.769 | 0.76 | 1.184 |
| Mean bias | -0.098 | -0.11 | -0.037 | 0.1 | -0.074 | 0.087 | 0.054 | -0.033 | -0.053 | 0.234 |
| Mean test length | 23.05 | 16.65 | 13.57 | 13.13 | 12.43 | 11.08 | 11.02 | 11.68 | 13.36 | 19.01 |
| Mean standard error | 0.346 | 0.318 | 0.317 | 0.316 | 0.298 | 0.291 | 0.292 | 0.294 | 0.295 | 0.406 |
| Proportion stop rule satisfied | 0.5 | 0.84 | 0.87 | 0.9 | 0.95 | 1 | 1 | 1 | 0.97 | 0.58 |
| Number of simulees | 100 | 100 | 100 | 100 | 100 | 100 | 100 | 100 | 100 | 100 |
| *SEM 0.387 (moderate precision)* | | | | | | | | | | |
| Mean Theta | -1.833 | -1.036 | -0.607 | -0.337 | -0.083 | 0.184 | 0.459 | 0.759 | 1.11 | 1.754 |
| RMSE | 0.877 | 0.995 | 1.114 | 1.307 | 0.746 | 0.687 | 0.702 | 0.754 | 0.808 | 1.176 |
| Mean bias | -0.096 | -0.023 | -0.022 | 0.155 | -0.021 | 0.118 | 0.076 | -0.042 | -0.026 | 0.239 |
| Mean test length (items) | 17.07 | 10.48 | 9.66 | 8.9 | 7.7 | 7.11 | 6.88 | 6.98 | 8.39 | 14.84 |
| Mean standard error | 0.398 | 0.387 | 0.383 | 0.386 | 0.372 | 0.367 | 0.372 | 0.372 | 0.37 | 0.455 |
| Proportion stop rule satisfied | 0.77 | 0.91 | 0.9 | 0.92 | 0.98 | 1 | 1 | 1 | 1 | 0.66 |
| Number of simulees | 100 | 100 | 100 | 100 | 100 | 100 | 100 | 100 | 100 | 100 |
| CAT=Computerized adaptive test; D=decile; DM=Diabetes Mellitus; RMSE=Root mean square error;  SEM=Standard error of measurement | | | | | | | | | | |

| **Additional File 24:** CAT simulation results for the Work domain at two precision levels | | | | | | | | | | |
| --- | --- | --- | --- | --- | --- | --- | --- | --- | --- | --- |
| *SEM 0.3 (high precision)* | | | | | | | | | | |
| **Measure** | **D1** | **D2** | **D3** | **D4** | **D5** | **D6** | **D7** | **D8** | **D9** | **D10** |
| Mean Theta | -1.833 | -1.036 | -0.607 | -0.337 | -0.083 | 0.184 | 0.459 | 0.759 | 1.11 | 1.754 |
| RMSE | 0.674 | 0.663 | 0.758 | 0.981 | 0.65 | 0.547 | 0.657 | 0.837 | 0.759 | 1.069 |
| Mean bias | -0.111 | -0.007 | -0.054 | -0.246 | -0.056 | -0.047 | -0.158 | -0.175 | -0.071 | 0.131 |
| Mean test length | 20.77 | 15.67 | 13.95 | 13.8 | 12.78 | 12.06 | 12.14 | 13.78 | 15.22 | 18.35 |
| Mean standard error | 0.347 | 0.305 | 0.304 | 0.308 | 0.296 | 0.294 | 0.294 | 0.295 | 0.297 | 0.39 |
| Proportion stop rule satisfied | 0.36 | 0.88 | 0.89 | 0.92 | 0.96 | 1 | 1 | 1 | 0.89 | 0.54 |
| Number of simulees | 100 | 100 | 100 | 100 | 100 | 100 | 100 | 100 | 100 | 100 |
| *SEM 0.387 (moderate precision)* | | | | | | | | | | |
| Mean Theta | -1.833 | -1.036 | -0.607 | -0.337 | -0.083 | 0.184 | 0.459 | 0.759 | 1.11 | 1.754 |
| RMSE | 0.771 | 0.676 | 0.809 | 1.089 | 0.626 | 0.581 | 0.682 | 0.852 | 0.835 | 1.075 |
| Mean bias | -0.065 | 0.033 | -0.036 | -0.312 | -0.134 | -0.111 | -0.219 | -0.254 | -0.066 | 0.153 |
| Mean test length (items) | 14.49 | 9.25 | 8.84 | 9.11 | 7.54 | 7.22 | 7.17 | 7.65 | 8.96 | 13.89 |
| Mean standard error | 0.398 | 0.38 | 0.376 | 0.383 | 0.375 | 0.374 | 0.376 | 0.376 | 0.374 | 0.439 |
| Proportion stop rule satisfied | 0.8 | 0.96 | 0.95 | 0.93 | 1 | 1 | 1 | 1 | 1 | 0.74 |
| Number of simulees | 100 | 100 | 100 | 100 | 100 | 100 | 100 | 100 | 100 | 100 |
| CAT=Computerized adaptive test; D=decile; RMSE=Root mean square error; SEM=Standard error of measurement | | | | | | | | | | |
